# Mangiferin as a Multilevel Modulator of Metabolic Syndrome: Current Evidence and Future Perspectives

**DOI:** 10.3390/metabo16070453

**Published:** 2026-06-27

**Authors:** Joan A. Ramos-Olvera, J. Martín Torres-Valencia, Mario I. Ortiz, Eduardo Fernández-Martínez, Carlos A. Gómez-Aldapa, Víctor Manuel Muñoz-Pérez, Raquel Cariño-Cortés

**Affiliations:** 1 Academic Area of Medicine, Instituto de Ciencias de la Salud, Universidad Autónoma del Estado de Hidalgo, Eliseo Ramirez Ulloa 400, Doctores, Pachuca 42090, Hidalgo, Mexico; ra281782@uaeh.edu.mx (J.A.R.-O.); mortiz@uaeh.edu.mx (M.I.O.); efernan@uaeh.edu.mx (E.F.-M.); victor_munoz9783@uaeh.edu.mx (V.M.M.-P.); 2 Academic Area of Chemistry, Instituto de Ciencias Básicas e Ingeniería, Universidad Autónoma del Estado de Hidalgo, Km. 4.5 Carretera Pachuca-Tulancingo, Mineral de la Reforma 42184, Hidalgo, Mexico; jmartin@uaeh.edu.mx (J.M.T.-V.); cgomeza@uaeh.edu.mx (C.A.G.-A.)

**Keywords:** mangiferin, metabolic syndrome, insulin resistance, obesity, microbiota, hyperlipidemia, hypertension

## Abstract

**Background/Objectives**: The obesogenic environment is a key factor in the rising prevalence of metabolic syndrome (MetS), a major global public health challenge. Mangiferin (Mgf) is a C-glycosylated xanthone, and its preventive and therapeutic potential stems from its multisystemic pharmacological profile. This review integrates findings on Mgf in the management of metabolic syndrome, aiming to identify gaps and propose prospective studies to enable the scaling up of Mgf for clinical applications. **Methods**: To this end, a literature search was conducted, the level of evidence was identified, and the available scientific information on Mgf in metabolic syndrome was synthesized from PubMed, Scopus, and Web of Science from 2016 to 2025. **Results**: Mgf improves overall metabolic dysfunction by activating the AMPK pathway, reduces inflammation by activating Nrf2, suppressing NF-κB, and decreasing pro-inflammatory mediators (COX-2, IL-6, NLRP3), modulates CB1 and PPAR receptors and other markers associated with obesity (adiponectin, leptin, resistin), as well as autophagy processes. However, most of the evidence comes from in silico, in vitro, and preclinical studies, with few clinical observations. **Conclusions**: This underscores the need to integrate studies on the correlation between the bioavailability of Mgf and norathyriol with the regulation of the microbiota, as well as their effects on metabolomic and epigenetic mechanisms in MetS.

## 1. Introduction

Metabolic syndrome (MetS) is a major public health challenge worldwide. It is characterized by a group of interconnected metabolic abnormalities, such as obesity, insulin resistance, dyslipidemia, and hypertension. These conditions coexist with persistent oxidative stress, which manifests as chronic low-grade inflammation over time [1,2]. The global prevalence of MetS is estimated at 25–35% in the adult population, reaching more than 40% in Latin America. It is estimated that at least 1.54 billion adults are affected worldwide. MetS significantly increases the risk of type 2 diabetes, cardiovascular disease, fatty liver disease associated with metabolic dysfunction, chronic kidney disease, cancer, and premature mortality [2,3].

In this context, the accumulation of visceral fat is considered a triggering factor in the pathophysiology of MetS. This leads to a systemic inflammatory profile due to the elevated production of pro-inflammatory cytokines, which disrupt insulin signaling [1,4]. These alterations are influenced by imbalances between caloric intake and expenditure in unhealthy lifestyles, particularly hypercaloric diets derived from ultra-processed foods coupled with sedentary behavior [5,6]. Several studies indicate that persistent activation of inflammatory pathways and redox imbalance contribute to endothelial dysfunction, impaired glycolipid homeostasis, and progression of cardiometabolic damage, reflecting the condition’s multisystemic nature [1,2,7].

Despite recent advances in the clinical management of MetS, pharmacological options remain limited in efficacy and adherence [8]. These drugs have moderate long-term efficacy and are associated with adverse effects that compromise adherence to treatment [9]. Available medications have limited capacity to modulate the underlying pathophysiological mechanisms of MetS, such as chronic inflammation and oxidative stress [2]. Additionally, since no single drug targets MetS as an integrated clinical entity, the therapeutic approach is polypharmacological, addressing its pathophysiological components simultaneously.

The American Diabetes Association (2024) [10] recommends metformin as the primary therapy for hyperglycemia. Sodium-glucose cotransporter 2 (SGLT2) inhibitors or glucagon-like peptide-1 (GLP-1) agonists are added based on cardiovascular risk. For dyslipidemia, the baseline therapy is statins, with the addition of fibrates if hypertriglyceridemia is present. For hypertension, the recommended therapy is angiotensin-converting enzyme (ACE) inhibitors or angiotensin II receptor blockers (ARBs), such as enalapril or losartan. For obesity, semaglutide is recommended in specific cases. For cardiovascular prevention, aspirin is recommended for patients at thrombotic risk. The American Heart Association (2026) has proposed a comprehensive approach aimed at reducing overall cardiovascular risk [11]. However, the prolonged and concomitant use of these drugs is associated with clinically significant adverse effects, including gastrointestinal disorders, an increased risk of hypoglycemia, peripheral edema, and electrolyte and muscle imbalances. This underscores the urgent need for complementary therapeutic strategies with more comprehensive mechanisms of action [12] and for multi-target, low-toxicity therapeutic agents.

In this context, there is a growing interest in using naturally occurring compounds as complementary therapeutic alternatives. Bioactive plant-derived compounds are being proposed as metabolic modulators with an improved safety profile. One promising candidate is mangiferin (Mgf). Mgf (1,3,6,7-tetrahydroxyxanthone-C2-β-D-glucoside) is a C-glycosylated xanthone found in various plant species, particularly in the leaves, roots, and fruits of mango (*Mangifera indica* L., Anacardiaceae), Chinese quince (*Pseudocydonia sinensis* (Thouin) C. K. Schneid.), zhimu (*Anemarrhena asphodeloides* Bunge), and honeybush tea (*Cyclopia genistoides*) [13]. Its preventive and therapeutic potential stems from its multifaceted pharmacological profile, which includes antidiabetic, antitumor, lipid metabolism regulation, cardioprotection, antihyperuricemic, neuroprotection, antioxidant, anti-inflammation, antipyretic, analgesic, antibacterial, antiviral, and immunomodulatory effects [14].

Taken together, the evidence suggests that Mgf can reconfigure energy and cellular metabolism in the context of metabolic imbalance [15,16,17,18,19]. This review aims to provide an integrated conceptual framework for using Mgf as a complementary therapeutic strategy in managing MetS. It explores the effects of Mgf on the endocrine-metabolic, inflammatory-oxidative, and lipid-metabolic axes of the condition, as well as its modulation of autophagy and the gut microbiota. This work is intended to guide future research and facilitate translation to clinical practice.

## 2. Materials and Methods

**Figure 1:**
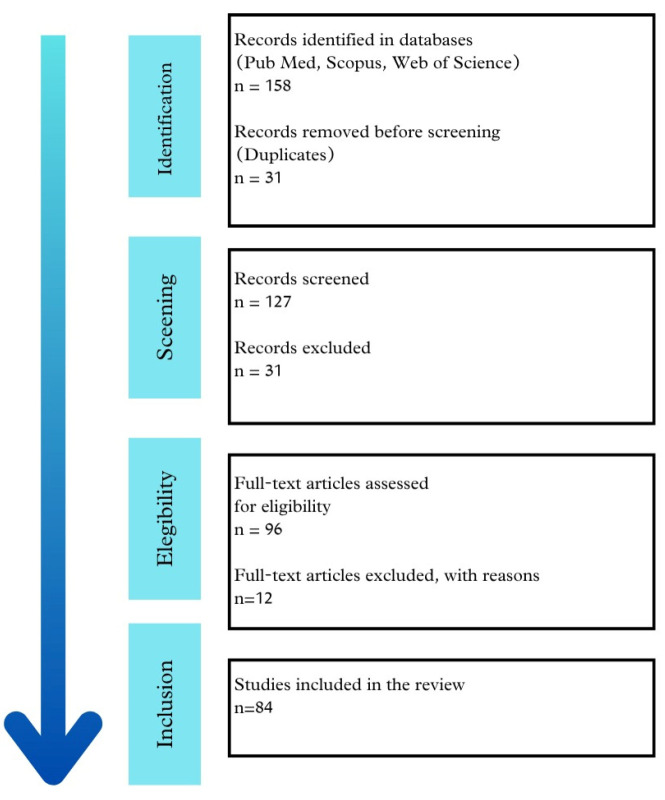
Simplified PRISMA diagram.

A literature search was conducted to find scientific evidence on the effects of Mgf on MetS. Articles published from 2016 to 2025 were searched in the PubMed, Scopus, and Web of Science databases. The search terms “mangiferin”, “mangiferina” and “C-glycosylated xanthone” were combined with keywords related to metabolic disorders and molecular mechanisms, including “metabolic syndrome,” “insulin resistance,” “obesity,” “microbiota,” “hyperlipidemia,” and “hypertension.” In vivo and in vitro studies, clinical observations in humans, review articles, and meta-analyses were included if they addressed standardized Mgf extracts or the biological, metabolic, or mechanistic effects of Mgf. Studies were excluded if they did not provide sufficient methodological information to permit critical appraisal of the reported findings. This included studies with unclear descriptions of experimental design, intervention protocols, outcome assessment, or analytical procedures. In addition, studies employing non-standardized extracts or lacking adequate characterization of their phytochemical composition were excluded. Other exclusion criteria included duplicate records, non-English publications, conference abstracts, editorials, and articles not directly related to the scope of this review. As this study was designed as a narrative review, a simplified PRISMA flow diagram (Figure 1) was used to provide transparency regarding the literature selection process. The final selection included studies considered most relevant and methodologically appropriate for addressing the objectives of this review.

## 3. Chemical and Pharmacokinetic Properties

**Figure 2:**
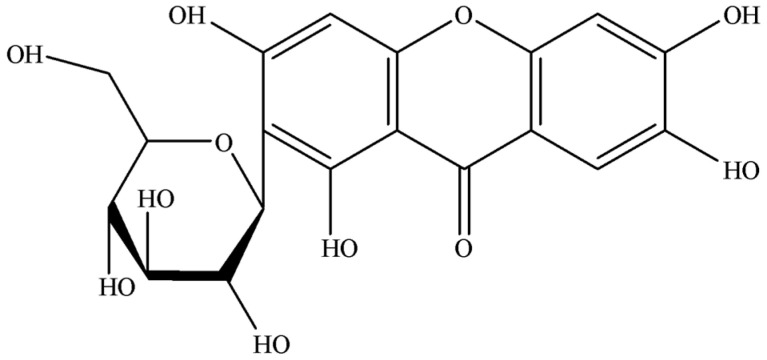
Chemical structure of mangiferin (1,3,6,7-tetrahydroxyxanthone-C2-β-D-glucoside).

Mgf (C_19_H_18_O_11_, 422 g/mol) is a crystalline compound characterized by a xanthone core with multiple reactive hydroxyl groups, particularly at positions C-2 and C-4, and a glucose unit linked by a β-glycosidic bond at C-7 (Figure 2). Mgf has a high melting point (274 °C) and a moderate partition coefficient (Log *P* = 2.73), reflecting its partially hydrophobic nature and low aqueous solubility (1.5 mg/mL) [20,21]. Mgf is slightly soluble in ethanol, sparingly soluble in methanol, and practically insoluble in diethyl ether, acetone, and *n*-hexane [22]. These properties limit its dissolution rate in biological fluids and constitute an initial obstacle to efficient oral absorption. Mgf is obtained mainly from *Mangifera indica*, with high concentrations found in young leaves (58.12 g/kg) and bark (18.33 g/kg). Ultrasound extraction has been shown to improve yield. However, the biopharmaceutical limitations of Mgf remain the main obstacle to its therapeutic use [23].

From a structure-activity relationship standpoint, the presence of multiple hydroxyl groups, a lactone carbonyl, and a stable C-glycosidic bond may account for Mgf’s pharmacological activity. However, these same structural features result in critical pharmacokinetic limitations, classifying Mgf as a low-solubility, low-permeability compound in class IV of the Biopharmaceutical Classification System (FDA, 2017) [13].

The poor transmembrane absorption of Mgf has been attributed to its C-glycosidic bond’s resistance to gastrointestinal hydrolysis, its low aqueous solubility, its reduced intestinal permeability, and its role as a substrate for P-glycoprotein, which further reduces its therapeutic concentration at sites of action [24,25]. These factors result in an extremely low bioavailability (~1.2%) after oral administration [13,26]. These limitations cause slow intestinal absorption, which primarily occurs by passive diffusion, as well as low *C*_max_ and AUC values [26]. Intestinal perfusion studies have shown that regional absorption follows the order duodenum > jejunum > colon > ileum, indicating an absence of active transport mechanisms [27].

**Figure 3:**
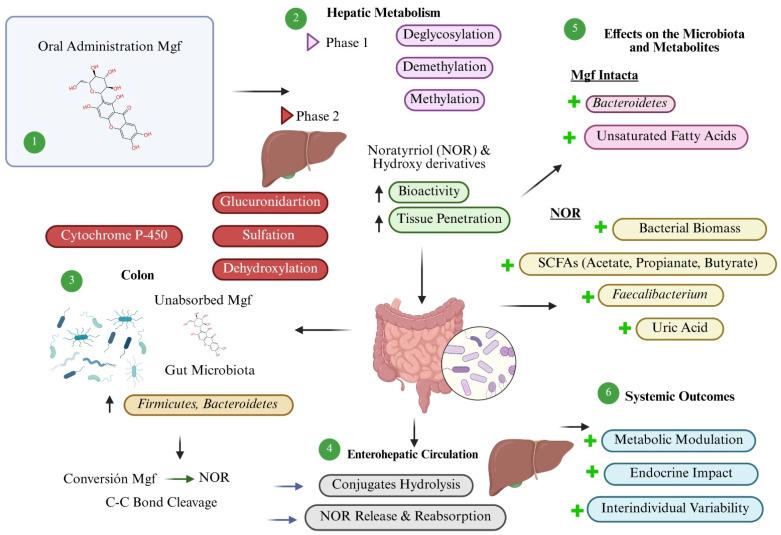
Sites of Mgf biotransformation. Mangiferin (Mgf) biotransformation is a sequential process involving the gut and liver. Mgf undergoes phase I metabolism (e.g., deglycosylation, dehydroxylation, and methylation), followed by transformations via methylation, glucuronidation, and sulfation. In gut microbiota-mediated metabolism, Mgf (a C-glucoside) is specifically hydrolyzed by C-glucosidases secreted by intestinal commensal bacteria, producing the aglycone noratiriol. This process is accompanied by the methylation of phenolic groups, which increases lipophilicity and absorption capacity. The metabolites then undergo phase II reactions in the liver, including glucuronidation, sulfation, and reglycosylation, as well as cytochrome P450-mediated modifications, such as dehydroxylation. These modifications fine-tune the metabolites’ biological activity. Finally, these compounds are eliminated through bile or urine. However, a fraction may be reprocessed by the intestinal microbiota, thereby establishing an enterohepatic cycle that prolongs its presence in the body.

Once absorbed, Mgf is extensively distributed in the tissues, reaching organs such as the liver, kidneys, lungs, heart, and brain, though generally at low concentrations. In vivo models detect peak levels primarily in the kidney and lung at around 6 h, suggesting relatively slow elimination and possible transient retention in highly perfused tissues [28] (see Figure 3). Its ability to cross specific biological barriers, such as the epidermal and dermal layers, has been documented, expanding its potential for topical and transdermal applications [29].

**Figure 4:**
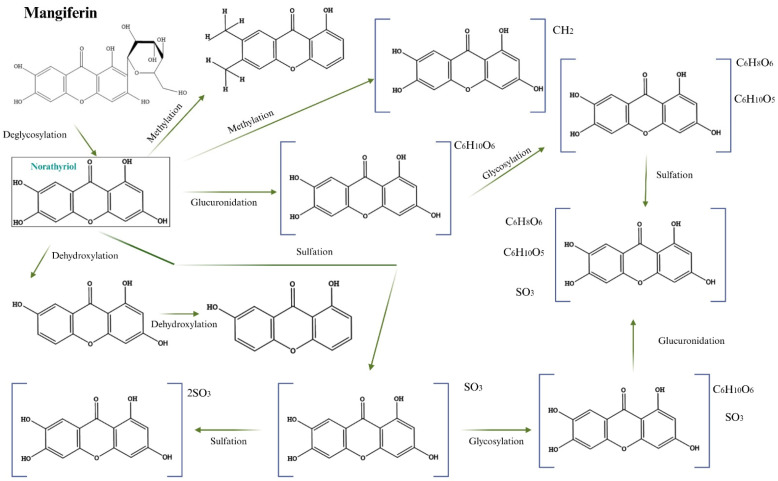
Pharmacokinetic process of Mgf. The process involves a multi-stage biotransformation through reactions including glycosylation, deglycosylation, methylation, glucuronidation, sulfation, and dehydroxylation, with the intestinal flora playing a key regulatory role in the early stages of metabolism.

Upon absorption, Mgf undergoes phase I reactions, including deglycosylation, dehydroxylation, and methylation. These reactions generate xanthone metabolites, such as norathyriol (NOR), as well as hydroxylated derivatives. Phase II reactions, including glucuronidation and sulfation, then occur. These processes significantly increase aqueous solubility and facilitate excretion (see Figure 4). Using Mgf, NOR, trihydroxyxanthone, and dihydroxyxanthone as templates, a rapid, efficient analytical strategy allowed for comprehensive profiling of Mgf metabolites in rats. A total of 67 Mgf metabolites (including Mgf itself) were detected. After oral administration, 29 metabolites were identified in plasma, 48 in urine, and 12 in feces. Additionally, six Mgf metabolites were detected in vitro in liver microsomes. Trihydroxyxanthones were detected for the first time in rat urine, along with novel bis-glucuronidation and sulfation metabolites derived from trihydroxyxanthones and dihydroxyxanthones. These findings demonstrate that Mgf metabolism involves deglycosylation, dehydroxylation, methylation, glucuronidation, and sulfation reactions. These transformations influence the drug’s systemic half-life and pharmacologically effective plasma concentration [30]. In this regard, Lin et al., 2020 [31] standardized the calcium salts of Mgf and validated the favorable pharmacokinetic profiles and the biochemical mechanisms in treating type 2 diabetes and non-alcoholic fatty liver disease (NAFLD). They found that the calcium salts of Mgf presented a better pharmacokinetic profile than Mgf, as they significantly favored its absorption, bioavailability, and efficacy.

Mgf has a wide safety margin. Acute and subchronic toxicity studies in rodents at doses up to 2 g/kg revealed no significant adverse effects or genotoxicity [32]. The hydrolysis of glucuronide and sulfate conjugates by gut microbiota promotes the release of aglycones, such as NOR. These aglycones can be reabsorbed via enterohepatic circulation, leading to secondary peaks in systemic concentration. The interaction between mangiferin, NOR, and the microbiota influences renal and fecal excretion, modulates microbial composition and function, and affects key metabolic pathways, such as short-chain fatty acid (SCFA) production and urate metabolism [33]. This complex interaction directly impacts the efficacy and safety of Mgf in long-term use.

## 4. Mechanisms of Action in Metabolic Syndrome

### 4.1. Insulin Resistance Level

There is varying scientific evidence regarding the efficacy and safety of Mgf, supporting the described mechanistic relationships and biological effects. The criteria presented in Table S1 (Supplementary file) are based on the hierarchy of evidence used in biomedical and pharmacological research, which classifies studies according to their level of experimental validation and clinical applicability.

**Figure 5:**
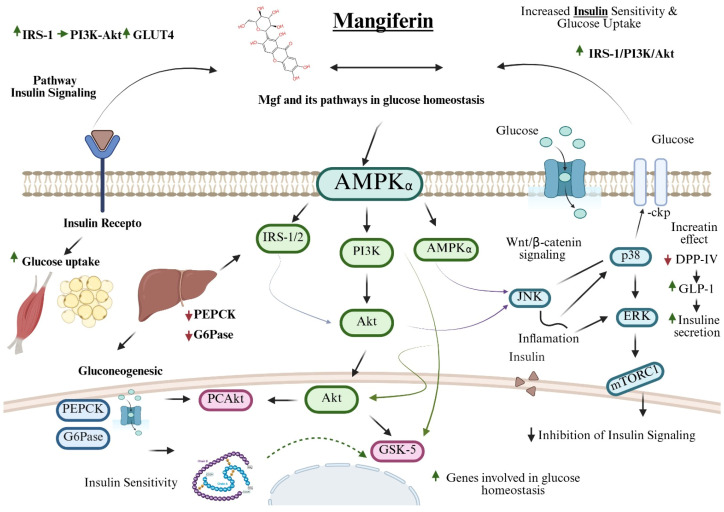
Molecular effects of Mgf on insulin resistance. Activation of AMPKα promotes insulin signaling via the IRS-1/PI3K/Akt axis, leading to increased glucose uptake through GLUT4 translocation and suppression of hepatic gluconeogenesis by downregulating PEPCK and G6Pase expression. Furthermore, AMPKα attenuates inflammation by inhibiting MAPK signaling pathways, including reducing the phosphorylation of JNK, p38, ERK1/2, and c-Jun, thereby improving the efficiency of insulin signaling. Mgf also modulates the Wnt/β-catenin pathway by inhibiting GSK-3β, thereby stabilizing and promoting the nuclear translocation of β-catenin. The β-catenin/TCF-LEF complex promotes the transcription of metabolic genes involved in glucose homeostasis. In addition, Mgf inhibits DPP-IV activity, leading to increased levels of incretins such as GLP-1, which enhances insulin secretion and further activates the IRS-1/PI3K/Akt signaling cascade.

First, Mgf showed significant inhibitory potential against α-amylase (IC_50_ = 9.72 mg/mL) and α-glucosidase (IC_50_ = 11.72 mg/mL), enzymes essential for the absorption of dietary carbohydrates. They delay the digestion of complex carbohydrates and reduce the rapid absorption of glucose, thereby preventing postprandial hyperglycemic spikes [34,35,36,37,38,39]. Additionally, they increase glucokinase (GK) activity by 20% above basal levels in HepG2 cell cultures and C2C1 myotubes [36,38,40]. This generates an optimized state of glucose metabolism, increases controlled energy flow, and converges on AMP-activated protein kinase (AMPK), the central node of the antihyperglycemic effect (see Figure 5). In this regard, several studies have shown that Mgf acts by regulating energy metabolism, promoting AMPK activation, modulating incretin secretion, and protecting pancreatic β cells. These actions contribute to improving insulin sensitivity and restoring glucose homeostasis in various experimental models with high-fat and high-sugar diets in mice, rats, and hamsters, as well as in genetically modified models (db/db, KK-Ay, and TSOD mice) [41,42,43,44]. Studies have shown that Mgf accelerated glycolytic flux, carbohydrate and lipid oxidation, and improved insulin sensitivity in skeletal muscle, liver, and heart [41,45,46,47]. Furthermore, Mgf increased hepatic expression of fibroblast growth factor 21 (FGF21) and elevated adiponectin levels, both of which promote insulin sensitivity and thermogenesis [48].

Mgf also improved mitochondrial oxidative capacity; this effect was associated with upregulation of mitochondrial gene transcription and increased expression and activity of succinate dehydrogenase (SDH). Furthermore, it helped prevent obesity-related muscle atrophy and sarcopenia in obese (fa/fa) and lean Zucker rats. These effects are related to increased mitochondrial biogenesis, driven by activation of the AMPK–PGC1α pathway [49]. The latter is considered a transcriptional coactivator that promotes mitochondrial biogenesis and, therefore, energy metabolism. In mesenchymal stem cells and adipose tissue, Mgf acted by suppressing PINK1–PRKN-mediated mitophagy. PINK1 (PTEN-induced kinase 1) detects dysfunctional mitochondria and recruits PRKN (Parkin), a ubiquitin ligase, which marks these mitochondria for selective mitophagy degradation to maintain functional mitochondrial biomass. It promotes the brown fat phenotype by increasing the expression of the uncoupling protein UCP1, enhancing selective mitochondrial quality and thermogenesis [50].

Mgf restores insulin signaling cascades, which are altered in insulin resistance. In particular, it enhances activation of the IRS-1/AKT/AMPK axis, increasing IRS-1 and phosphorylated AKT levels, thereby facilitating insulin signaling in the liver and aortas of Wistar rats with dexamethasone-induced insulin resistance [43]. Furthermore, it ameliorates insulin resistance by inhibiting NF-κB-mediated inflammation and activating the AMPK signaling pathway in vitro [51], and acts as a dual activator of PPARγ and GLUT4, increasing mRNA expression to enhance insulin sensitivity and glucose uptake in pancreatic tissue [52]. Additionally, it inhibits protein tyrosine phosphatase 1B (PTP1B), a negative regulator of insulin signaling [53], and reduces renal interstitial fibrosis in diabetic nephropathy models by inhibiting the PTEN/PI3K/Akt signaling pathway [54].

Furthermore, in silico and in vivo studies have shown that it participates in the regulation of incretins. Both mango leaf extract and Mgf stimulated the secretion of glucagon-like peptide-1 (GLP-1) by activating the MAPK (p-ERK1/2, p-p38, p-c-Jun) and Wnt (β-catenin, GSK-3β, Axin1) signaling pathways in the human cell line NCI-H716 L [55]. In rats, it significantly reduced fasting blood glucose, HbA1c, and lipids, while increasing serum GLP-1 levels and inhibiting dipeptidyl peptidase IV (DPP-IV), showing effects comparable to those of sitagliptin and vildagliptin [56,57]. In the pancreas, Mgf induced islet hyperplasia and robust β-cell proliferation, while inhibiting apoptosis, even in aging models [42]. Regeneration and restoration of beta cell function occurred through modulation of cell cycle enzymes, such as cyclins (D1/D2 and CDK4), and the reduction in senescence marker expression (p16INK4a and p27Kip1) [58]. In this context, in male Wistar Hannover and male SD rats with STZ-induced diabetes and an alveolar bone defect, a beneficial effect was observed, decreasing apoptosis, inflammation, and oxidative stress, and promoting bone regeneration [59,60]. Topical application of 1% or 2% Mgf gel improved wound healing in a streptozotocin-induced diabetes model. These effects reflected increased growth factors (EGF, FGF, TGF-β, VEGF), PI3K signaling, and depletion of NF-κB and TNF-α [61]. In Swiss albino mice with alloxan-induced diabetes, oral administration of a hydro-alcoholic extract of *M. indica* for 7 days improved glucose levels, lipid profile, and pancreatic oxidative stress [62]. Another complication in diabetes is wound healing in infectious contexts. In this regard, it has been shown that Mgf nanoparticles (Rh@Ag-MFG) and a biohybrid nanorobot (MF@DeMEV/SA-MNP) loaded with mangiferin were effective in improving wound closure rate, bacterial counts, ROS levels, and signaling pathways such as PI3K–Akt, TGF-β, IL-6, Nrf2, and CD31 [63,64].

In other endocrine contexts, Mgf improves hormonal balance in polycystic ovary syndrome models. It reduces hormonal imbalance by lowering the LH/FSH ratio and insulin resistance. At the ovarian level, it has anti-apoptotic effects by inhibiting mitochondrial release of Cyt, an event that activates the pro-apoptotic cascade, thereby preventing activation of Caspase-3, the caspase responsible for apoptosis [65].

### 4.2. Inflammatory-Oxidative Level

Mgf exerts a regulatory effect on inflammation and systemic oxidative stress through complementary mechanisms. It has broad-spectrum antioxidant capacity stemming from its structural characteristics: four hydroxyl groups on a xanthone core, two of which interact with electrophilic species, forming two phenoxyl radicals. These are stabilized by resonance, allowing them to intercept and neutralize free radicals [32], including via chelating ferrous ions. These events inhibit the subsequent formation of hydroxyl radicals in the Fenton reaction and in lipid peroxidation [66]. On the one hand, it directly neutralizes reactive oxygen species; on the other, it strengthens endogenous antioxidant systems and modulates inflammatory signaling pathways. These effects help restore cellular homeostasis and limit the damage associated with glycolipotoxicity in metabolic syndrome [41,66,67,68].

**Figure 6:**
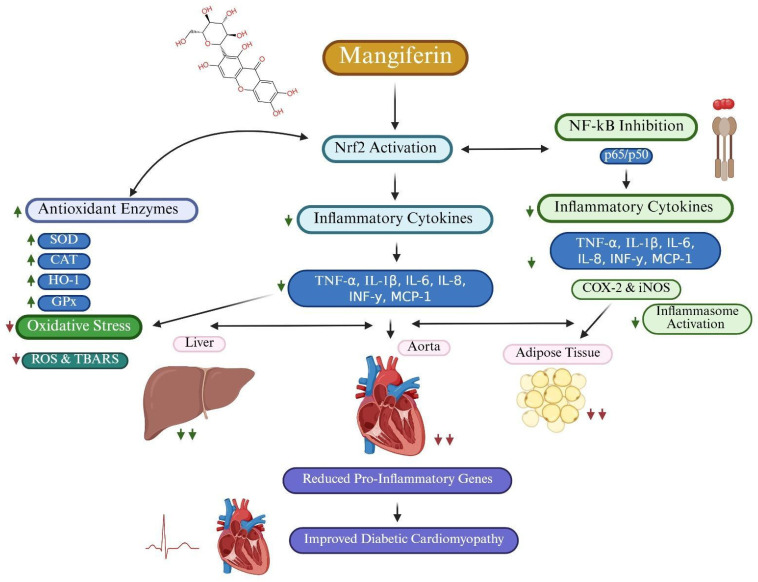
**Effects of Mgf on oxidative-inflammatory stress**. Mgf exerts a protective effect against oxidative stress and inflammation by activating the Nrf2/ARE pathway, increasing the expression of endogenous antioxidant enzymes (SOD, CAT, GPx, and HO-1), and reducing ROS and TBARS levels. Simultaneously, it inhibits the NF-κB (p65/p50) pathway and NLRP3 inflammasome activation, thereby decreasing the expression of pro-inflammatory mediators. These effects in the liver, aorta, and adipose tissue contribute to the improvement of diabetic cardiomyopathy. SOD, superoxide dismutase; CAT, catalase; GPx, glutathione peroxidase; HO-1, heme oxygenase-1; ROS, reactive oxygen species; TBARS, thiobarbituric acid reactive substances; Nrf2, erythroid-related nuclear factor 2 (ERGF2). AREs, antioxidant response elements; NF-κB, nuclear factor kappa B; NLRP3, NOD-like receptor with pyrin domain 3.

Mgf has demonstrated a cytoprotective effect against oxidative damage by reducing lipid peroxidation, as evidenced by the strengthening of the endogenous enzymatic antioxidant system (SOD, CAT, GSH-Px) and the decrease in oxidative stress markers (TBARS and ROS) in various experimental models of diabetes, as well as in clinical studies in overweight and obese humans (Figure 6) [69,70].

Mgf has been shown to activate Nrf2, a transcription factor that regulates the antioxidant response to pro-oxidant stimuli, which promotes the dissociation of Nrf2 from its inhibitory protein Keap1 and its subsequent translocation to the nucleus, where it binds to antioxidant response elements (AREs), increasing the expression of superoxide dismutase (SOD), catalase (CAT), glutathione peroxidase (GPx), and heme oxygenase-1 (HO-1) [17,66].

Mgf also inhibits the activation of the transcription factor NF-κB (p65 and p50 subunits), which reduces the transcription of pro-inflammatory genes and limits the activation of the inflammasome (hepatic NOD-like receptor family pyrin domain-containing protein 3 or NLRP3) [19,43,44,67,71,72]. As a result, a decrease in pro-inflammatory cytokines and enzymes, such as TNF-α, IL-1β, IL-6, IL-8, IFN-γ, and MCP-1, as well as COX-2 and iNOS, is observed in the liver, aorta, and adipose tissue [48,49,73]. In this context, Mgf significantly improved diabetic cardiomyopathy induced in Sprague-Dawley rats fed a high-fat streptozotocin diet by deactivating the NF-κB pathway (reducing p65 nuclear translocation), thereby inhibiting the release of inflammatory cytokines and reducing the accumulation of reactive oxygen species (ROS). It also suppressed the formation of advanced glycation end products (AGEs) and decreased RAGE mRNA and protein expression. These actions led to decreased myocardial damage markers and reduced collagen aggregation in the heart [47]. Additionally, in another study with Wistar rats with diabetes and ischemia–reperfusion-induced cardiac damage, Mgf was observed to significantly improve cardiac function and preserve myocardial architecture. It worked by inhibiting the AGE–RAGE axis, JNK, and p38 MAPK phosphorylation, while increasing ERK1/2 expression, thereby preventing oxidative stress, inflammation, and apoptosis [74]. In in silico molecular docking and in vitro studies, Mgf and its derivatives reduced the activity of the polyol pathway, specifically aldose reductase, which could imply beneficial effects on diabetic retinopathy and diabetic pulmonary fibrosis [75,76,77]. Thus, Mgf exhibited protective cardiometabolic effects, improving endothelial function, reducing vascular damage and vascular wall thickening, promoting the recovery of blood flow in ischemic limbs, and decreasing insulin resistance.

Hemodynamic and vascular alterations are among the main complications of MetS. In this context, under physiological conditions, activation of AKT (also known as Protein Kinase B, PKB) promotes eNOS activity and NO generation, thereby favoring vasodilation. However, in contexts of insulin resistance and oxidative stress, phosphatase and tensin homolog (PTEN) inhibits AKT, leading to reduced NO bioavailability and endothelial dysfunction. In this regard, in a C57BL/6J mouse model of induced vascular damage, Mgf negatively regulated PTEN expression by increasing p-Akt and p-eNOS expression, thereby restoring NO bioavailability and reducing ROS production, thus preventing thickening of the abdominal aortic wall [78,79]. Additionally, the restorative effects of repair and vasodilation have been demonstrated in experimental models of ischemia–reperfusion combined with streptozotocin-induced diabetes, where a mango extract containing 0.5% Mgf showed upregulation of eNOS and intercellular adhesion molecule-1 (ICAM-1), a marker of endothelial activation, while inducible nitric oxide synthase (iNOS) was downregulated [80]. Likewise, in models of hyperuricemia and hypertension, Mgf has inhibitory effects on systolic blood pressure (SBP), NO, eNOS, C-reactive protein (CRP), and intercellular adhesion molecule-1 (ICAM-1) [81].

Metabolic syndrome acts as an amplifier of vascular damage, facilitating mechanisms that promote abdominal aortic aneurysm (AAA), as both share common pro-inflammatory mechanisms (activation of NF-κB, IL-6, and TNF-α). In AAA, Signal Transducer and Activator of Transcription 3 (STAT3) promotes Krüppel-like factor 5 (KLF5). This promotes the proliferation of vascular smooth muscle cells (VSMCs), inflammation, and dedifferentiated vascular remodeling. These events contribute to weakening of the vascular wall and the development of aneurysms. In this context, high-dose magnesium phosphate (Mgf) in ApoE (-/-) mice reduced the incidence of AAAs and elastin degradation, stabilized the contractility of vascular smooth muscle cells (VSMCs), and inhibited apoptosis [82].

The monocyte chemoattractant protein-1 (MCP-1) axis and its receptor CCR2 in monocytes are among the main regulators of renal inflammation and biomarkers of kidney damage progression, especially in the context of metabolic syndrome. In this regard, in spontaneously hypertensive rats (SHRs), Mgf alleviated renal inflammatory injury by inhibiting the MCP-1/CCR2 signaling pathway, reducing IL-6 and TNF-α levels, and promoting IL-10. It did not significantly reduce systolic blood pressure but did protect against kidney damage [73].

Additionally, in a multi-omics analysis combined with systems pharmacology, treatment with the monosodium salt of Mgf provided renoprotective effects in rats with STZ-induced diabetic nephropathy (DN) by alleviating insulin resistance (IR)-induced systemic renal inflammation and podocyte IR. These mechanisms correlated primarily with Mgf-induced inhibition of the MAPK/NF-κB axis and activation of the p-IRS-1 (Tyr608)/p-PI3K/p-Akt axis in the kidneys of DN rats. Mgf had a beneficial effect on renal ferroptosis in STZ-induced diabetic nephropathy rats by increasing mevalonate-mediated antioxidant capacity (GPX4 axis and FSP1/CoQ10) and weakening pro-apoptotic lipid generation in the kidneys mediated by ACSL4 [83].

In parallel, Mgf promotes glucose uptake and enhances insulin signaling by activating AMPK through phosphorylation. Activation of this kinase helps modulate endoplasmic reticulum (ER) stress, attenuating the overactivation of the unfolded protein response (UPR) by regulating proximal sensors such as IRE1α and PERK/eIF2α, as well as downstream pro-apoptotic effectors such as CHOP [84]. In this context, the protective effect of Mgf against ER stress has been shown in models of endothelial dysfunction induced by diets rich in saturated fats in rodents, where its action, mediated in part by AMPK, is associated with an improvement in endothelial sensitivity to insulin, the reduction of oxidative stress and the inhibition of NLRP3 inflammasome activation [67,68,85,86]. Accordingly, in a dexamethasone-induced insulin resistance model, Mgf decreased aortic expression of pro-inflammatory and vasoconstrictor mediators such as endothelin-1 (ET-1), VCAM-1, JNK, and NF-κB, as well as VEGF, while increasing eNOS expression and prostacyclin (PGI2) levels, suggesting a comprehensive improvement in endothelial function [43].

Additional evidence has indicated that Mgf inhibits the activation of the transcription factor NF-κB (p65 and p50 subunits), which reduces the transcription of pro-inflammatory genes and limits the activation of the NLRP3 inflammasome [19,43,44,67,72,87]. As a result, the production of pro-inflammatory cytokines such as TNF-α, IL-1β, IL-6, IL-8, IFN-γ, and MCP-1 decreases, as does the expression of pro-inflammatory enzymes such as COX-2 and iNOS in tissues such as the liver, aorta, and adipose tissue [48,49,73].

On the one hand, it can directly neutralize reactive oxygen species; on the other, it strengthens endogenous antioxidant systems and modulates inflammatory signaling pathways, thereby reducing the pro-inflammatory state and oxidative damage. However, a recent study observed a possible pro-inflammatory effect in a carrageenan-induced acute inflammation model at doses of 50–100 mg/kg of Mgf isolated from *Hedysarum neglectum*. While in silico models predicted anti-inflammatory and antidiabetic effects, in vivo tests showed pro-inflammatory activity and no significant reduction in glucose or cholesterol. These discrepancies are attributed to the molecule’s extremely low oral bioavailability (1.2%) and poor transmembrane permeability [88]. In this regard, Lin et al. (2019) [89] found that the Mgf content was much higher after administration of decoctions of *Rhizoma Anemarrhenae* than with the monomer, suggesting the importance of identifying the coexisting components in the extract that may enhance Mgf bioavailability.

### 4.3. Level of Lipid Metabolism

Mangiferin exerts multifaceted metabolic effects, including regulating intracellular signaling pathways, inhibiting intestinal lipid uptake, suppressing lipogenic processes, and stimulating fatty acid oxidation. Mgf acts locally in the digestive tract through non-competitive, reversible inhibition of pancreatic lipase (IC50 = 82.31 µmol/L) by binding to an allosteric site on the enzyme. In mice, it reduced the absorption of dietary lipids, increasing fecal lipid excretion by 52.5%, thereby contributing to reduced body weight gain and attenuated hyperlipidemia [18].

**Figure 7:**
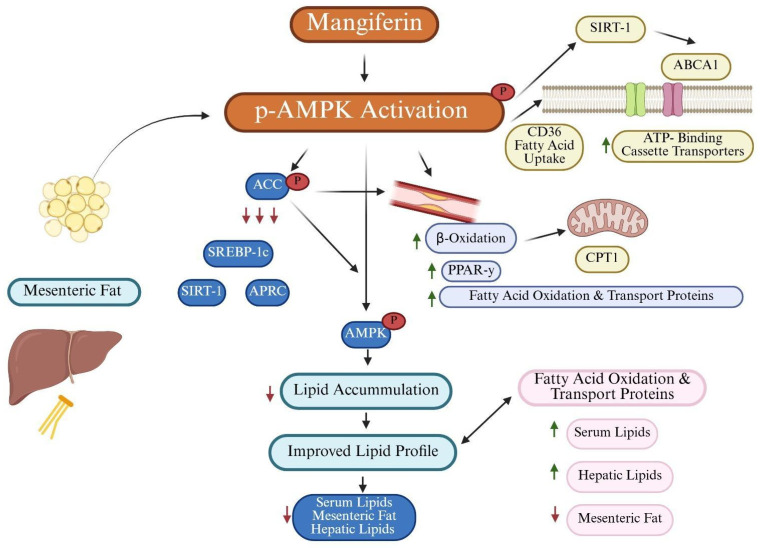
Effects of Mgf and its metabolites on the lipid profile. Mangiferin activates the AMPK pathway, inhibiting lipogenesis by downregulating ACC, SREBP-1c, FAS, and PPAR-γ, partly through the SIRT-1/AMPK axis. Simultaneously, it promotes fatty acid oxidation by increasing PPARα, CPT1, and CD36, thus favoring the energy use of lipids. Furthermore, it increases the expression of the ABCA1 and ABCG1 transporters, improving cholesterol handling. Together, these effects reduce lipid accumulation and improve the metabolic profile. AMPK: AMP-activated protein kinase; ACC: Acetyl-CoA carboxylase; SREBP-1c: Sterol regulatory element-binding protein 1c; FAS: Fatty acid synthase; PPAR-γ: Peroxisome proliferator-activated receptor gamma; SIRT-1: Sirtuin 1; PPARα: Peroxisome proliferator-activated receptor alpha; CPT1: Carnitine palmitoyltransferase 1; CD36: Fatty acid translocase; ABCA1: ATP-binding cassette transporter A1; ABCG1: ATP-binding cassette transporter G1.

Systemic actions occur predominantly in key metabolic tissues, including the liver, adipose tissue, and skeletal muscle [64,90]. In this regard, the SIRT-1/AMPK/SREBP-1c signaling pathway constitutes a metabolic axis that regulates lipid homeostasis in the liver. According to the research of Li J. et al. (2018) [71], Mgf acts on this pathway primarily through its active metabolite NOR to combat hepatic steatosis and metabolic disorders in male C57BL/6 and KK-Ay mice. Activation of Sirtuin-1 (SIRT-1) by NOR promoted the deacetylation of hepatic kinase B1 (LKB1), increasing its cytoplasmic-to-nuclear ratio; this, in turn, promoted the phosphorylation of AMP-activated protein kinase (AMPK) at residue Thr172. AMPK activates, phosphorylates, and inactivates acetyl-CoA carboxylase (ACC), reducing malonyl-CoA production and the disinhibition of CPT1 (carnitine palmitoyltransferase 1), facilitating the entry of fatty acids into the mitochondria for β-oxidation. In addition, activated AMPK directly phosphorylates Ser372 of SREBP-1c, preventing its nuclear translocation and inhibiting the transcription of lipogenic genes, such as fatty acid synthase (FAS) (Figure 7).

Further studies have shown that Mgf significantly reduces mesenteric fat accumulation and serum and liver lipid levels. This effect is associated with activation of the AMPK signaling pathway, as evidenced by increased phosphorylation, leading to inhibition of lipogenic processes at both the post-translational and transcriptional levels. In particular, AMPK phosphorylates and directly inhibits ACC and reduces the expression of lipogenic factors such as fatty acid synthase (FAS) and PPAR-γ, possibly through the modulation of transcriptional regulators such as SREBP-1c. Additionally, Mgf increases the expression of PPARα and proteins involved in fatty acid transport and oxidation, such as CD36 (fatty acid translocase) and CPT1 (carnitine palmitoyltransferase 1). It is worth noting that although CD36 facilitates fatty acid uptake, its induction in this context appears to be functionally coupled to an increase in CPT1-mediated mitochondrial β-oxidation, which favors lipid use as an energy source rather than storage. Taken together, these tissue-context-dependent mechanisms contribute to reducing lipid accumulation, improving the lipid profile, and attenuating the development of metabolic disorders such as hyperlipidemia and hepatic steatosis [45,90,91].

In adipocytes, magnesium phosphate of Mgf and mango extracts have demonstrated a significant capacity to counteract obesity-related mechanisms and uncontrolled adipogenesis by inhibiting the differentiation of 3T3-L1 preadipocytes into mature adipocytes. This process is achieved through the downregulation of PPARγ, considered the master regulator of adipocyte differentiation, as well as other adipogenic markers, including the transcription factor inducing lipogenic genes, such as Sterol Regulatory Element-Binding Protein-1 (SREBP-1), the enzyme of de novo lipogenesis FAS, and DGAT1/2, enzymes that catalyze the last steps of lipogenesis [50,92]. Similarly, Jack et al. 2017 [93] evaluated the effect in this model, finding that Mgf decreased intracellular lipid accumulation, lipid content, lipolysis (glycerol release), and mRNA expression of HSL (hormone-sensitive lipase) and UCP3 (uncoupling protein 3). At a systemic level, the extract with Mgf administered to Wistar rats with HFD-induced obesity reduced abdominal fat accumulation and improved systemic inflammation (increased IL-10, reduced TNF-α). It acts by positively regulating PPAR-γ and LPL (promoting fat oxidation and uptake) and negatively regulating FAS (inhibiting de novo lipogenesis) [94]. Mgf increases the expression of the PPARγ and FALDH genes in adipose tissue, which are essential for maintaining glucose homeostasis and lipid metabolism [95].

Additionally, in male golden Syrian hamsters with HFD-induced hyperlipidemia, Mgf intervened by enhancing the oxidation of free fatty acids (FFAs), increasing 3-hydroxybutyrate and acetate levels, and reducing the accumulation of triglycerides (TG) and total cholesterol (TC) via the AMPK/PPARα pathway [96,97]. Likewise, in male C57BL/6J mice, both Mgf and its amino acid derivative (L-phenylalanine methyl ester), synthesized to improve liposolubility (85x) and potency, significantly improved lipid profile and adipogenesis biomarkers [98].

Mgf also participates in cholesterol homeostasis, acting as an anti-atherogenic factor. It has been shown to increase the expression of ATP-binding cassette transporters ABCA1 and ABCG1, which facilitate the removal of excess cholesterol from peripheral tissues and macrophages and its transport to the liver for subsequent excretion. It suppresses hepatic lipogenesis and accelerates lipid degradation via the AMPK pathway in Apoe -/- mice and RAW264.7 cells [90]. Furthermore, it improves cholesterol homeostasis by decreasing the expression of genes involved in cholesterol synthesis and transport (hmgcr, sqle, srebf2, sp1, and ldlr) and by increasing the expression of genes involved in cholesterol catabolism (cyp7a1) in primary catfish hepatocytes and NCTC 1469 mouse hepatocytes [99].

In addition, Vimang (an aqueous extract of *Mangifera indica* L. stem bark containing 16% Mgf) was added to the diet of male LDL receptor-deficient mice (LDLr-/-), a genetic model for familial hypercholesterolemia prone to atherosclerosis. It significantly reduced plasma cholesterol by 15% and liver cholesterol by 20%, likely through enhanced fecal excretion of steroids. It increased plasma total antioxidant capacity by 10% and markedly reduced (50%) ROS production in spleen mononuclear cells. However, the short treatment duration was insufficient to significantly reduce the size of early atherosclerotic lesions [100]. Brito et al. [101] evaluated adipogenic markers in response to a cafeteria diet in Wistar rats and macrophage cultures. The findings indicated that mango leaf extract (250 mg/kg) had a greater effect than Mgf (40 mg/kg) in decreasing TNF-α and CB1 expression and increasing adiponectin during co-treatment and that MAN appeared to act as a CB1 receptor agonist.

Several experimental and clinical studies have shown that Mgf administration significantly reduces serum levels of triglycerides (TG), total cholesterol (TC), and LDL cholesterol, while increasing HDL cholesterol [15,43,56,70,102]. In this regard, Pinneo et al. [103] conducted a randomized crossover study with two dietary interventions that included a mango-based snack. Favorable biomarkers of glucose, insulin, and adiponectin, as well as satiety, were observed. Another study demonstrated that oral supplementation with 650 mg of a proprietary blend (LI12542F6) of *S. indicus* flower and *M. indica* bark extracts (in a 2:1 ratio) or a placebo for 56 consecutive days, combined with a 4-day-per-week resistance training program in athletes, increased muscle endurance, mitochondrial activity, testosterone, and the mTOR/eNOS signaling pathway [104].

### 4.4. Autophagy and Cellular Homeostasis

Mgf activates autophagy in macrophages via specific molecular cascades. In the AMPK/mTOR axis, Mgf stimulates AMPK phosphorylation and inhibits mTORC1 complex synthesis (which is a physiological inhibitor of autophagy), thereby initiating the formation of double-membrane autophagosomes. Mgf also increases the expression of proteins essential for autophagic flux, including LC3-II, p62, Beclin 1, ATG5, and ATG7 in in vitro models using the rat pancreatic β-cell line INS-1 and murine macrophage RAW264.7 cells [90,105]. In a similar manner, Mgf reduces the formation of atherosclerotic plaques. A combination of Mgf and the synthetic Mgf ligand T0901317 promotes lipid degradation via autophagy (lipophagy) and increases the expression of smooth muscle α-actin (α-SMA), thereby increasing collagen content and stabilizing the plaques to prevent rupture [90].

**Figure 8:**
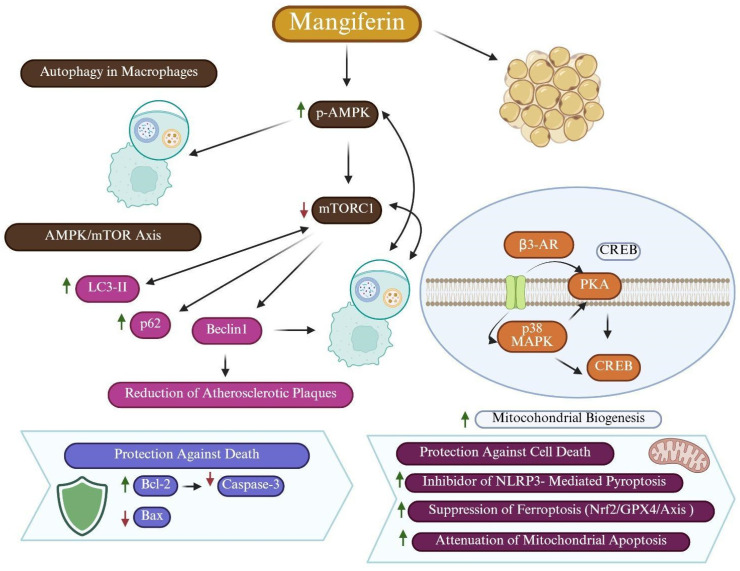
Mgf stimulates autophagy and cellular homeostasis. Activation of the AMPK/mTOR pathway promotes an increase in autophagic markers: LC3-II, p62, and Beclin 1 in cultured macrophages and pancreatic cells. Furthermore, Mgf promotes lipophagy, selective mitophagy, and reduces apoptosis in cardiomyocytes, thereby enhancing mitochondrial efficiency.

Interestingly, under glycolipotoxic conditions, the autophagic flux is typically blocked. However, Mgf can restore this process by promoting autophagosome turnover [106]. This effect is supported by changes in autophagic markers, such as LC3II, p62, Beclin1, Atg5, and Atg7, which indicate efficient degradation of damaged cellular material [106,107]. This AMPK-dependent activation significantly reduces body weight gain and fat accumulation in the liver and adipose tissue. It improves glucose and lipid metabolism by inhibiting inflammatory responses mediated by macrophages (reducing M1 ATM macrophages and TNF-α) and by activating autophagy in the liver (increasing ATG7 and FGF21 levels) [48,90] (Figure 8).

Mgf also participates in the selective regulation of mitochondrial degradation, or mitophagy. In adipose tissue, Mgf promotes a brown fat phenotype by suppressing mitophagy through activation of the β3-AR-dependent signaling pathway, which activates PKA, p38 MAPK, and CREB. These events lead to increased mitochondrial biogenesis, improved respiratory function (OCR), and increased UCP1 expression. Increased expression of key transcriptional regulators such as PGC-1α, TFAM, NRF1, and NRF2 is associated with the latter process. These factors regulate mitochondrial replication and cellular respiratory capacity [50,108].

Furthermore, Mgf protects cells against programmed cell death induced by Ang-II-induced cardiomyocyte hypertrophy by increasing Bcl-2 expression and decreasing Bax expression, thereby enhancing cell survival [72,74]. Mgf has also been shown to reduce the activation of caspase-3 and the release of cytochrome c (Cyt c), two key events in the apoptotic cascade, in a polycystic ovary syndrome (PCOS) model [65]. In vascular smooth muscle cells, a high dose of Mgf reduced the incidence of abdominal aortic aneurysms and elastin degradation. It stabilized VSMC contractility and inhibited apoptosis by preventing the translocation of the transcription factor STAT3 [82].

Mgf exhibits multiple cytoprotective effects by modulating various regulated pathways of cell death. These effects include inhibiting NLRP3-mediated pyroptosis, suppressing ferroptosis via activation of the Nrf2/GPX4 axis, and reducing mitochondrial apoptosis. Mgf exerts a multi-target regulatory effect by inhibiting the NF-κB pathway, thereby suppressing NLRP3 inflammasome activation. It effectively blocks both canonical (Caspase-1-mediated) and noncanonical (Caspase-11-mediated) pyroptosis cascades, ultimately preventing cleavage of GSDMD and subsequent cell membrane rupture. These effects demonstrate Mgf’s potential to modulate cell death in metabolic and inflammatory diseases [90,109].

### 4.5. Level of the Gut Microbiota

The gut microbiota plays a key role in the action of Mgf, converting the poorly absorbed parent compound into the active metabolite NOR. NOR exhibits greater intestinal permeability and biological activity [67,110,111]. Bacterial phyla, such as *Bacteroidetes*, and specific strains, such as *Bacillus* KM7-1, have been identified as capable of performing this biotransformation. Following oral administration, Mgf levels in the liver are lower than those of its metabolites, indicating that NOR is the final mediator of numerous biological processes [18].

Diabetes and other metabolic alterations can affect the composition of gut bacteria. Mgf exhibited low oral bioavailability (1.71% in normal rats and 0.80% in diabetic rats) due to a significant first-pass effect. The maximum concentration was lower and the clearance half-life was significantly longer in diabetic rats than in normal rats [111,112].

In experimental models of PCOS, Mgf administration induced favorable changes in the gut microbiota. It increased the relative abundance of *Firmicutes* and *Bacteroidetes* while reducing the abundance of *Proteobacteria* and *Actinobacteria*. Mgf also promoted the growth of beneficial genera, such as *Blautia*, *Coprococcus*, and *Roseburia* (Firmicutes) and *Bifidobacterium* (Actinobacteria), while reducing potentially pathogenic genera commonly associated with chronic inflammation, including *Pseudomonas*, *Sutterella*, *Prevotella*, and *Helicobacter* [65,113].

The interaction between Mgf and the gut microbiota may depend on bacterial communication mechanisms, such as quorum sensing. In this regard, a mixture containing Mgf (20 or 60 mg/kg) and *Lactobacillus reuteri* (SML) was more effective at reducing fasting blood glucose and improving glucose tolerance than either component alone. The mixture improved insulin sensitivity and increased beneficial gut flora (probiotics) and the quorum-sensing molecule AI-2, which is present in feces [114].

Mgf-induced changes in the gut microbiota are associated with metabolic and hormonal improvements in the host. For instance, higher levels of beneficial bacteria, such as *Lactobacillus*, are linked to more balanced levels of follicle-stimulating hormone (FSH) and estradiol (E2), as well as lower levels of testosterone and insulin [65]. These results suggest that Mgf should be considered a microbial prodrug whose efficacy and safety depend on intestinal biotransformation processes and enterohepatic circulation. These findings imply that chronic use of Mgf should be approached with caution and highlight the importance of integrating microbiota and metabolism analysis into future therapeutic evaluations.

## 5. Challenges and Future Directions

Although Mgf has low oral bioavailability, this limitation does not necessarily preclude its therapeutic efficacy. In silico, in vitro, and in vivo evidence indicates that Mgf favorably modulates energy metabolism by activating AMPK, the master sensor of cellular energy status, promoting catabolic states such as glucose uptake, glycolysis, lipolysis, and autophagy, and inhibiting anabolic processes such as glycolipotoxicity, oxidative stress, and the pro-inflammatory state generated by MetS.

Mgf belongs to a class of polyphenolic compounds that share similar phamacokinetic limitations and biological activities with other phytochemicals such as curcumin, resveratrol, quercetin, and berberine. For example, curcumin has shown potent antioxidant, anti-inflammatory, and anticancer effects despite its extremely low systemic bioavailability, owing to rapid metabolism and poor absorption [115]. Similarly, resveratrol exhibits low oral bioavailability due to extensive first-pass metabolism, but maintains cardiometabolic and neuroprotective effects in experimental and clinical models [116]. Berberine also demonstrates clinically relevant hypoglycemic and lipid-lowering effects despite minimal plasma exposure, largely attributed to gut-mediated mechanisms and active metabolites [117]. Furthermore, flavonoids such as quercetin are limited by poor absorption and rapid metabolism, but still exhibit important anti-inflammatory and antioxidant properties. While all these compounds exhibit pleiotropic biological activities, including antioxidant, anti-inflammatory, and metabolic regulatory effects, they differ substantially in terms of mechanistic characterization, pharmacokinetic behavior, and clinical validation. These examples highlight that pharmacological efficacy can depend not only on high systemic exposure but also on local gastrointestinal activity, tissue accumulation, interactions with the microbiota, and bioactive metabolites derived from the administered parent compound.

While the overall body of evidence suggests beneficial effects of Mgf on individual components of MetS, the interpretation of the findings should account for methodological heterogeneity across studies. Differences in experimental conditions, formulations, doses, treatment durations, and outcome assessments may partially explain the variability observed across reports. In this context, experimental evidence and clinical observations are more closely related to effective exposure at the target site than to plasma concentration alone. Therefore, even if Mgf has low oral bioavailability, its biological effects observed in preclinical models can be explained by sufficient local accumulation, metabolic activation, or high affinity for its molecular targets.

To date, human studies have primarily focused on pharmacokinetic characterization and exploratory efficacy assessments using Mgf-enriched mango leaf extracts. Available evidence confirms the poor systemic exposure of Mgf after oral administration. The first human pharmacokinetic study by Hou et al. 2012 [115] demonstrated nonlinear pharmacokinetics and low plasma concentrations following oral dosing, whereas the recent study by Fuentes-Ríos et al. 2025 [116] showed that formulation optimization through a water-soluble monosodium derivative significantly increased systemic exposure, highlighting pharmaceutical formulation as a key strategy for overcoming one of the major translational barriers of Mgf. However, these investigations were not conducted within a conventional drug-development framework and therefore cannot be considered formal phase II or phase III efficacy trials. Likewise, recent human pharmacokinetic studies have improved the understanding of Mgf absorption and metabolism but have not yet established therapeutic dosing regimens for MetS.

Interventions involved oral Mgf at doses ranging from 5 to 422 mg/kg for durations of 11 days to 16 weeks, using models that induce metabolic syndrome-independent alterations [17,41,66,79]. The induction of type 2 diabetes, hypertension, and obesity has demonstrated modulation of signaling pathways related to insulin resistance, lipid metabolism, inflammation, autophagy, and the gastrointestinal microbiota. All these integrated processes lead to the restoration of homeostasis in in vitro and preclinical models.

These findings highlight that pharmacological activity at the preclinical level would imply an average dose of 250 mg/kg, equivalent to the human dose calculated by normalizing body surface area to approximately 40.5 mg/kg (≈2.8 g/day for a 70 kg adult), suggesting that the effective preclinical dose could translate into a relatively high intake in humans. The doses of Mgf evaluated in humans have ranged from 150 mg/day for 12 weeks, promoting a cytoprotective effect by restoring energy balance, as well as antioxidant and anti-inflammatory effects [41]. Although clinical evidence remains scarce, several registered interventional studies have evaluated Mgf-containing mango leaf extracts in humans, particularly regarding cognitive function, mood, cerebral blood flow, and muscle efficiency parameters [116,117]. Nevertheless, most studies have involved standardized extracts rather than purified Mgf, and additional randomized clinical trials are needed to establish its therapeutic efficacy, safety profile, and optimal dosing regimens.

Mgf encapsulation strategies could improve both preventive and therapeutic peformance by increasing its solubility, stability, and intestinal absorption, thereby increasing its effective bioavailability. This makes it essential to re-evaluate its toxicological safety. On the other hand, controlled and targeted release systems could facilitate higher local concentrations at the site of action, while simultaneously reducing systemic exposure. These advantages could also allow for a reduction in the high doses required in preclinical studies.

Therefore, future studies could focus on large-scale, multicenter clinical trials, standardized dosing regimens, long-term safety assessments, and the identification of validated biomarkers that can reproducibly demonstrate physiological benefits. In parallel, harmonizing extraction procedures, quality control standards, and the chemical characterization of Mgf-rich products will be essential to ensure batch-to-batch consistency and facilitate regulatory approval under different health legislations.

The pharmaceutical form also presents challenges. While Mgf exhibits remarkable pharmacological activities in vitro and in animal models, its low oral bioavailability, limited permeability, and rapid metabolism continue to restrict its clinical development. Therefore, future efforts should prioritize pharmaceutical optimization through advanced delivery systems, such as nanoparticles, liposomes, nanoemulsions, phospholipid complexes, and controlled-release formulations. In addition, comprehensive pharmacokinetic and pharmacodynamic studies are required to establish exposure–response relationships, identify active metabolites, and define therapeutic windows in humans.

Finally, the limitations of this review include the possibility of omitting some studies and of not including relevant findings published in languages other than English. Furthermore, the literature selection focused primarily on articles from peer-reviewed JCR journals, without systematically incorporating books or scientific or academic literature not subject to traditional bibliographic control (ISBN, ISSN), which may have resulted in incomplete coverage of literature types.

## 6. Conclusions

Mgf is a promising multisystem modulator that has been shown to affect energetic metabolism, inflammation, oxidative stress, dyslipidemia, and autophagy. It also has emerging potential as a regulator of epigenetics and the gut microbiota. However, Mgf’s low oral bioavailability compromises its efficacy, warranting the development of advanced formulations.

Most of the available evidence stems from in silico, in vitro, and preclinical studies. However, clinical evidence is limited, underscoring the need for further human research to confirm the biological effects of Mgf. Robust clinical studies are lacking, and no pharmacokinetic studies or controlled phase II and III clinical trials have been conducted in humans with MetS.

Current clinical evidence suggests that Mgf is generally safe and well tolerated at the doses evaluated to date; however, the limited number of human studies and the heterogeneity of dosing regimens preclude the establishment of a standardized therapeutic dose. Additional clinical studies are therefore required to define its therapeutic window, optimal dosage, and long-term safety.

At the mechanistic level, significant gaps remain in epigenetic and epigenomic evidence, and comprehensive studies of Mgf’s effects on the gut microbiota are needed. Future Mgf studies will focus on overcoming its limitations and optimizing its effects.

Due to its pleiotropic profile, Mgf is a promising multi-target therapy for MetS with good potential for synergistic effects with established drugs, such as metformin or GLP-1 agonists.

New strategies, including modulation of the gut microbiota and development of nanoformulations to address low bioavailability, are being explored. In silico models will also enable the prediction of new molecular targets and metabolic interactions, paving the way for the therapeutic and/or preventive use of Mgf.

## Data Availability

No new data were created or analyzed in this study.

## Supplementary Materials

The following supporting information can be downloaded at: https://www.mdpi.com/article/10.3390/metabo16070453/s1, Table S1: Preclinical and clinical evidence of mangiferin and standardized extracts in metabolic syndrome.

## Abbreviations

The following abbreviations are used in this manuscript:

AAA	Abdominal Aortic Aneurysm
ACE	Angiotensin-Converting Enzyme
ACSL4	Acyl-CoA Synthetase Long Chain Family Member 4
AGEs	Advanced Glycation End Products
AKT	Protein Kinase B
AMPK	AMP-Activated Protein Kinase
ARBs	Angiotensin II Receptor Blockers
AREs	Antioxidant Response Elements
AUC	Area Under the Curve
Axin-1	Axis Inhibition Protein 1
CAT	Catalase
C2C1	Creatine Kinase MM Isoform (CK-MM)
CDK4	Cyclin-Dependent Kinase 4
Cmax	Maximum Plasma Concentration
CoQ10	Coenzyme Q10
COX	Cyclooxygenase
Cyt	Cytochrome
DPP-IV	Dipeptidyl Peptidase IV
eNOS	Endothelial Nitric Oxide Synthase
ERK	Extracellular Signal-Regulated Kinase
FGF	Fibroblast Growth Factor
FSH	Follicle-Stimulating Hormone
GLP-1	Glucagon-Like Peptide 1
GLUT4	Glucose Transporter Type 4
GSH-Px	Glutathione Peroxidase
GSK-3β	Glycogen Synthase Kinase 3 Beta
HbA1c	Glycated Hemoglobin
HepG2	Human Hepatocellular Carcinoma Cell Line
HO-1	Heme Oxygenase 1
ICAM	Intercellular Adhesion Molecule
IFN-ɣ	Interferon Gamma
IL	Interleukin
iNOS	Inducible Nitric Oxide Synthase
IP	Intraperitoneal
IRS-1/2	Insulin Receptor Substrate 1 and 2
JNK	c-Jun N-terminal Kinase
KLF	Krüppel-Like Factor
LH	Luteinizing Hormone
MCP	Monocyte Chemoattractant Protein
MetS	Metabolic Syndrome
Mgf	Mangiferin
mRNA	Messenger Ribonucleic Acid
mTOR1	Mechanistic Target of Rapamycin Complex 1
NFⲕ-B	Nuclear Factor Kappa B
NLRP3	NLR Family Pyrin Domain Containing 3
Nrf2	Nuclear Factor Erythroid 2-Related Factor 2
p16INK4a	Cyclin-Dependent Kinase Inhibitor 2A
p38	p38 Mitogen-Activated Protein Kinase
p27Kip1	Cyclin-Dependent Kinase Inhibitor 1B
PGC1	Peroxisome Proliferator-Activated Receptor Gamma Coactivator 1
PINK1	PTEN-Induced Kinase 1
PKB	Protein Kinase B
PPAR	Peroxisome Proliferator-Activated Receptor
PRKN	Parkin RBR E3 Ubiquitin Protein Ligase
PTEN	Phosphatase and Tensin Homolog
PTP1B	Protein Tyrosine Phosphatase 1B
RAGE	Receptor for Advanced Glycation End Products
ROS	Reactive Oxygen Species
SCFA	Short-Chain Fatty Acids
SDH	Succinate Dehydrogenase
SGLT2	Sodium–Glucose Cotransporter 2
SOD	Superoxide Dismutase
TSOD	Total Superoxide Dismutase
STAT	Signal Transducer and Activator of Transcription
TBARS	Thiobarbituric Acid Reactive Substances
TNFɑ	Tumor Necrosis Factor Alpha
UCP-1	Uncoupling Protein 1
Wnt	Wingless-Related Integration Site
