# Supplementary material for: Mangiferin as a Multilevel Modulator of Metabolic Syndrome: Current Evidence and Future Perspectives
Source: Metabolites. 2026 Jun 27;16(7):453. doi: 10.3390/metabo16070453 (PMC13413803; doi:10.3390/metabo16070453)
Supplement: Supplementary file 1 [file metabolites-16-00453-s001.zip › metabolites-4355671-supplementary.pdf]

Tabla S1. Preclinical and clinical evidence of mangiferin and standardized extracts in metabolic syndrome.

Ref.	Study Model/Population	Intervention (concentration/Doses/ duration	Mechanism of Action	Level of evidence
[49]	Zucker rats (fa/fa) phenotype and lean Zucker as controls.	15 mg/kg BW/day p.o. Mgf into gelatin pellets/ 8 weeks.	↓Inflammatory cytokines (IFN-γ, IL-1β, IL-6, IL-10).<br>↓Muscle atrophy (sarcopenia)<br>↑Muscle oxidative capacity<br>↑Mitochondrial biogenesis (SDH and Citrate Synthase activity).	IIId
[43]	36 male Wistar rats (3 months old, weighing 180–250 g).	Oral administration of Mgf (25, 50, or 100 mg/kg) once daily for 14 days. Dexamethasone (1 mg/kg) was injected from day 8 to 14 to induce insulin resistance.	↓Insulin resistance (restoring the IRS1/AKT signaling pathway in the liver and aorta)<br>↑Antioxidant, anti-inflammatory, and Lipidemic effects<br>↓NLRP3/NF-κB activation<br>↓Oxidative damage<br>↑eNOS and PGI2 elevation	IIId
[70]	Overweight female volunteers (n = 31), aged 25–45 years, with a BMI of 25.0–29.9.	Consumption of 1 g mango peel powder (MPP) sachet taken twice daily, 30 minutes before meals, for 84 days.	↓ LDL, ↑ HDL, ↓ ROS	II
[101]	In vitro: THP-1 human macrophage cell line.<br>In vivo: Male Wistar rats fed a cafeteria diet (CD) to induce adipogenesis.	Compounds: Ethanolic extract of Mangifera indica leaves (EMI) and isolated Mgf.<br>Doses: 250 mg/kg for EMI and 40 mg/kg for Mgf administrated by gavage.<br>Duration: Post-treatment (5 days of intervention after 7 days of CD) and Co-treatment (8 days of intervention along side the diet).	EMI ↓TNF-α and CB1 expression<br>↑Adiponectin during co-treatment.<br>Mgf appeared to act as a CB1 receptor agonist.<br>EMI and Mgf were non-cytotoxic<br>↓Adipogenesis: EMI>Mgf.	IIIb
[75]	In silico molecular docking and in vitro enzymatic assay using healthy male Wistar Albino rats (200–250 g) to provide eye lenses for AR inhibition.	Mgf concentrations ranging from 12.5 to 300 μg/ml for the in vitro assay. In silico simulations targeted the human aldose reductase (2R24).	↓ AR,<br>↓Poliol pathway<br>↓ Diabetic retinopathy	IIIc
[90]	Male ApoE−/− mice (8 weeks old) fed a high-fat diet (HFD) and in vitro cultures of RAW264.7	Mgf (200 mg/kg/day) and/or the LXR ligand T0901317 (T0) (1 mg/kg/day) administered via oral gavage for 16 weeks. In vitro macrophages were treated	↑Cholesterol efflux<br>↓Plaque formation<br>↑Hyperglyceridemia<br>↑AMPK<br>↑fatty liver	IIIb

Ref.	Study Model/Population	Intervention (concentration/Doses/ duration	Mechanism of Action	Level of evidence
	(murine macrophage) and THP-1 (human monocytic) cell lines.	with Mgf (50 μM) and T0 (1 μM).	↓lipogenesis	
[82]	In vivo: Male apolipoprotein E-deficient (ApoE-/-) mice; in vitro: Primary rat and human aortic smooth muscle cells (RASMCs/HASMCs).	In vivo: Low-dose (10 mg/kg/d) or high-dose (50 mg/kg/d) mangiferin via intraperitoneal injection for 28 days. In vitro: 25 μM mangiferin.	↓AAA incidence<br>↓Elastin<br>↓VSMC contractility<br>↓Apoptosis<br>↓STAT3 translocation<br>↓KLF5 activity	IIIb
[76]	In silico/Computational model using Human Aldose Reductase (PDB ID 2ikh) and simulation for Rattus norvegicus targets.	Virtual screening and molecular dynamics (MD) comparison of Mgf and six derivatives (HMF, IMF, NMF, GMF, MFG, NRT).	↓ Aldose reductase<br>↓ Aldose reductase allosteric	V
[112]6/27/2026 3:54:00 PM	In vitro gastrointestinal digestion and colonic fermentation using feces from six healthy human volunteers (three men and three women, aged 20–24). It also utilized a Caco-2 human intestinal epithelial cell monolayer model.	Caco-2 Transport: 200 μM of Mgf for 180 minutes.<br><br>Fermentation: Final concentration of 10 mg/mL of MGF incubated for 96 hours.	↓Permebility (< 2%)<br>↑Microbiota utilization (46.37% by 96 hours)<br>↑Beneficial genera (Bacteroides and Bifidobacterium)<br>↓Harmful genera (Sutterella and Prevotella).<br>↑Unsaturated fatty acid synthesis (oleic and linolenic acids)<br>↑longevity regulation	IVa
[100]	Four-month-old male LDL receptor-deficient mice (LDLr −/−), a genetic model for familial hypercholesterolemia prone to atherosclerosis.	Vimang® (an aqueous extract of Mangifera indica L. stem bark containing 16% Mgf) added to the diet at 2 g/kg to achieve a daily oral dose of 250 mg/kg. The treatment lasted 2 weeks, with a cholesterol-enriched diet introduced during the second week.	↓Cholesterol by 15% and liver cholesterol by 20%<br>↑Fecal excretion of steroids<br>↑Antioxidant, ↓ ROS	IIId
[108]	Primary cell culture using mouse bone-	Cells were stimulated with 1 μg/mL of Lipopolysaccharide	↓Pro-inflammatory cytokines TNF-α, IL-6, IL-1β, and IL-18.	IVa

Ref.	Study Model/Population	Intervention (concentration/Doses/ duration	Mechanism of Action	Level of evidence
	marrow-derived macrophages (BMDMs)	(LPS) to induce cell pyroptosis. Mgf at doses of 10, 50, or 100 μg/mL/24h.	↓mRNA levels of Caspase-1, Caspase-11, and GSDMD.<br>↓NLRP3, Caspase-1, Caspase-11, and NF-κB, and decreased the cleavage of GSDMD into its active N-terminal.	
[41]	This is a literature review that synthesizes data various models, including in vitro studies (cell lines like 3T3-L1, HepG2, and C2C12 myotubes), preclinical animal models (high-sucrose or high-fat diet (HFD) fed mice, rats, and hamsters; genetic models like db/db, KK-Ay, and TSOD mice), and human clinical data (overweight patients with hyperlipidemia).	In vitro: 10 μM to 200 μM.<br>In vivo (Animals): Oral or i.p. doses ranging from 10 mg/kg to 400 mg/kg.<br>Human: 150 mg/day for 12 weeks.	↓ TG, ↓ FFA, ↑ HDL<br>↑Insulin sensitivity, ↑Glucose oxidation<br>↑AMPK<br>↑Lipid catabolism, ↑Carbohydrate oxidation<br>↓Lipogenesis<br>↓ROS<br>↓NF-κB↓ MAPK (anti-inflamatory)	IVb
[37]	In vitro enzymatic assays and in vivo male Wistar rats with STZ-induced diabetes.	Mgf-loaded solid lipid nanoparticles (MG-SLNs). In vivo dose: 40 mg/kg BW.	↓MG-SLNs, α-amylase, α-glucosidase, BGL (12 h)<br>↑Bioavailability (↑lymphatic, ↓ first-pass, ↑permeation)	IIIb
[79]	A review focusing on five polyphenols, with specific sections on Mgf using endothelial cell lines (HUVECs, RRCECs), rodent models of vascular injury and diabetes (HFD mice, STZ rats), and human clinical trials.	In vitro: 20 μM (HUVECs).<br>In vivo (Animals): 5 mg/kg to 400 mg/kg BW.<br>Human: 150 mg/day for 12 weeks.	↑ Endothelial function<br>↓ Vascular injury and wall thickening<br>↑ Blood flow<br>↓ Insulin resistance<br>↓ AGE/RAGE<br>↑ PTEN/Akt/eNOS<br>↓ Angiogenes HIF-1α and VEGF	IVb

Ref.	Study Model/Population	Intervention (concentration/Doses/ duration	Mechanism of Action	Level of evidence
[77]	The research combined human lung surgical resection specimens (20 patients), male C57BL/6 mice with STZ-induced diabetes, and human umbilical vein endothelial cells (HUVECs)	In vivo: Mice received 20 or 60 mg/kg of Mgf orally every 3 days for 4 weeks. In vitro: HUVECs were treated with 10 or 50 μM Mgf for 72 hours under high glucose conditions	⬇ Fibrosis (EndMT)<br>⬆ SIRT3<br>⬆ FoxO3⬇ Injury and fibrosis	II
[110]	Male Sprague-Dawley (SD) rats, including both normal rats and those with alloxan-induced diabetes.	Mgf was administered as a single dose: oral (10 mg/kg) and intravenous (2 mg/kg).	⬇Bioavailability (1.71%→0.80%)<br>⬇ First-pass, diabetic<br>⬇ Cmax<br>⬆ t 1/2	IIId
[98]	In vitro: 3T3-L1 preadipocytes, a widely used model for researching fat metabolism and adipocyte differentiation.<br>In vivo: Obesity was induced in mice using a high-fat diet (HFD) and high-sugar drinking water for a period prior to treatment.	In vivo: Male C57BL/6J mice (6 weeks old).<br>Compound: G1, a novel Mgf amino acid derivative (L-phenylalanine methyl ester) synthesized to improve liposolubility and potency.<br>In vitro: Concentrations ranging from 0 to 500 μM were used for screening, with G1 showing an IC_{50} of 39.13 μM in 3T3-L1 cells.<br>In vivo: Intraperitoneal (i.p.) administration at 30 mg/kg (low dose, G1-I) and 60 mg/kg (high dose, G1-II). Administered every 2 days for a total of 8 weeks.	⬆Liposolubility (85x),<br>⬇ Weight/fat<br>⬇TG/TC/LDL, ⬆ HDL<br>⬇Lipid accumulation, ⬇FAS<br>⬇Adipogenesis (G0/G1), ⬇ROS	IIIb
[96]	Male golden Syrian hamsters (*Mesocricetus auratus*) with HFD-induced hyperlipidemia.	Mgf administered orally via gavage at 150 mg/kg BW once daily for 8 weeks.	⬇ Disorders/triglycerides<br>⬆ Oxidation (3 HB/acetate)<br>⬇ Glucometabolism (lactate/alanine)	IIId

Ref.	Study Model/Population	Intervention (concentration/Doses/ duration	Mechanism of Action	Level of evidence
[47]	Male Sprague-Dawley rats with Diabetic Cardiomyopathy (DCM) induced by a high-fat diet for 4 weeks followed by a low dose of streptozotocin (35 mg/kg).	Mgf administered at 20 mg/kg daily by oral gavage for 16 weeks.	↓ Nf-kB<br>↓ Cytokines/ROS<br>↓ AGEs/RAGE<br>↓ Collagen	IIId
[73]	Spontaneously hypertensive rats (SHRs).	Mgf administered at doses of 10, 20, or 40 mg/kg/day for 8 weeks consecutively.	↓ MCP-1/CCR2<br>↓ IL-6<br>↓ TNF-α<br>↑ IL-10<br>↓ Kidney damage	IIId
[66]	A comprehensive review covering cell cultures (e.g., HL-60 leukemia, U87 glioma, human umbilical vein endothelial cells (HUVECs)), animal models (STZ-induced diabetic rats, HFD mice, lung carcinoma mice), and human studies (overweight hyperlipidemic patients).	In vitro: 1 µg/mL to 100 µg/mL; 10 µM to 200 µM.<br>In vivo (Animals): 10 mg/kg to 400 mg/kg BW.<br>Human: 150 mg/day for 12 weeks.	↓ Oxidative damage<br>↓Radicals, iron, inflammation (NF-κB/IRAK1/MAPK)<br>↑ Nrf2<br>↑ Antioxidant defense<br>↑ AMPK	IVb
[93]	In vitro study using 3T3-L1 murine pre-adipocytes and mature adipocytes.	Intervention (Concentration/Doses/Duration): Treatment with polyphenol-enriched fractions (F1–F4), where F3 is predominantly Mgf (44.28 g/100 g). Doses ranged from 1, 10, 50, to 100 µg/mL. Pre-adipocytes were treated daily for 8 days during differentiation, while mature adipocytes were treated for 24 hours.	All fractions:<br>↓ Lipids<br>↑ HSL<br>↑ UCP3<br>↑ Oxidation	IVa
[55]	Docking studies	Inhibitory potential of 27 compounds.	↑ HSL<br>↑ UCP3	IIIa

Ref.	Study Model/Population	Intervention (concentration/Doses/ duration	Mechanism of Action	Level of evidence
	In vitro study using the human NCI-H716 L-cell line<br>Male Sprague-Dawley rats with type 2 diabetic rat model induced (fructose 10% w/v/12 weeks and STZ 30 mg/kg i.p.)	In vitro: L-cells were treated with 5, 10, or 30 μg/mL of mango leaf extract (MLE) for 2 hours. In vivo: Diabetic rats were administered 40 mg/kg MLE ((165.67 ug/g Mgf) orally for 12 weeks.	↑ Oxidation<br>↑ GLP-1 (MAPKs/β-catenin)<br>↓ Glucose/HbA1c/lipids<br>↑ GLP-1<br>↓ DPP-IV	
[44]	In vitro THP1-derived macrophages and HEK293 reporter cells.	Mgf treatment at 10 and 25 μM under a hyperglycemic microenvironment (HGM).	↑ Nrf2/AKT<br>↑ Antioxidant<br>↓ NF-κB/NLRP3<br>↓ Cytokines/ROS<br>↑ Macrophage function	IVa
[78]	In vivo: Male C57BL/6J mice with high-fat diet (HFD)-induced vascular injury.<br>In vitro: Human Umbilical Vein Endothelial Cells (HUVECs) stimulated by oxidized low-density lipoprotein (ox-LDL).	In vivo: Mgf at 5 mg/kg and 20 mg/kg mixed with HFD for 12 weeks.<br>In vitro: Mgf pretreatment at 5, 10, and 20 μM for 12 hours.	↓ PTEN<br>↑ Akt/eNOS<br>↑ NO/ ↓ ROS<br>↓ Lipids/inflammation	IIIb
[59]	Male Wistar Hanover rats with STZ-induced diabetes and a created alveolar bone defect. It also used rat Bone Mesenchymal Stem Cells (BMSCs).	Local implantation of Mgf-loaded PLGA scaffolds into the bone defect, with drug content ranging from 4.71% to 12.79% w/w; evaluations were conducted up to 21 weeks.	Diabetic bone repair:<br>↓ Apoptosis BMSCc<br>↓ Inflammation<br>↓ Oxidative stress	IIIb
[71]	KK-Ay mice used for in vivo validation and HepG2 human liver cells for in vitro mechanism studies.	Mgf administered orally at 100 or 200 mg/kg/day for 4 weeks. In vitro cells were treated with MGF or its metabolite norathyriol (up to 100 μM).	↑ AMPK<br>↑ p-SREBP-1c<br>↓ Synthesis<br>↑ Oxidation<br>↑ Lipolysis	IIIb
[89]	Male SD rats, categorized into healthy groups and	A single intragastric dose of 35 mg/kg of Mgf (as a monomer, or	↑ Liver<br>↑ Kidneys<br>↓ Pancreas	IIId

Ref.	Study Model/Population	Intervention (concentration/Doses/ duration	Mechanism of Action	Level of evidence
	Type 2 Diabetic (T2DM) groups (induced by high-fat diet and STZ).	within Rhizoma Anemarrhenae or herb pair decoctions).	↓ Intestine	
[31]	Sprague-Dawley rats of both genders (200-230 g) used for pharmacokinetic and T2DM/NAFLD disease models.	Mgf calcium salt (MCS) was given in single doses (60, 240, 960 mg/kg) or multiple doses (240 mg/kg/day for 7 days). In the disease model, doses were 120, 240, or 480 mg/kg.	MCS<br>↑Absorption/Bioavailability<br>↓T2DM/NAFLD (TCA, fatty acids, tryptophan)	IIId
[46]	Male C57BL6/J mice fed either a chow diet or a high-fat diet (HFD) for 16 weeks. It also used C2C12 myotube cell cultures.	Oral Mgf at 400 mg/kg body weight mixed in food for 16 weeks. Cell cultures were treated with 500 μM for 4 hours.	↑ Glycolytic flux<br>↑ Mitochondrial oxidative capacity<br>↑ Mitochondrial gene transcription<br>↑ SDH expression/activity<br>↑ ATP	IIIb
[105]	In vivo model using male C57BL/6J mice fed a high-fat diet (HFD) and an in vitro model using the rat pancreatic β-cell line INS-1.	In vivo: 25 mg/kg/day of Mgf administered intraperitoneally for 2 weeks. In vitro: 25, 50, and 100 μM of Mgf for 24 hours under high glucose/palmitic acid (HG/PA) conditions.	↓ Glucolipotoxicity<br>↑ AMPK/ ↓ mTOR<br>↑ Autophagy<br>↓ Apoptosis/ ↑ insulin/ ↑ glucose/ ↓ injury	IIIb
[61]	Male Sprague-Dawley rats with STZ-nicotinamide-induced type-2 diabetes and an excisional wound.	Topical application of 1% or 2% Mgf gel (or 1% silver sulphurdiazine as control) daily for 21 days.	↓ Wound size<br>↑ Skin thickness<br>↑ PI3K<br>↑ Growth factors<br>↓ NF-κB/ TNF-α	IIId
[113]	48 male Sprague–Dawley rats (180 ± 20 g) with STZ-induced type 2 diabetes.	A synbiotic (SML) composed of Mgf (20 or 60 mg/kg) and *L. reuteri* 1–12 (diluted from 5×10 ^{10}<br>CFU/mL) was administered daily for 6 weeks	↑ FGB<br>↑ Glucose Tolerance<br>↑ Insulin sensitivity<br>↑ Probiotics<br>↑AL-2	IIId
[36]	Combined in silico screening, in vitro assays (recombinant GK enzyme, HepG2 cells, and C2C12 myotubes), and in vivo male db/db diabetic mice.	In vitro: Mgf concentrations from 0 to 1 mM for 24 hours. In vivo: 200 mg/kg/day administered orally for 8 weeks.	↑ Glucose consumption/In vivo	IIIa

Ref.	Study Model/Population	Intervention (concentration/Doses/ duration	Mechanism of Action	Level of evidence
[17]	Systematic review that included 13 human intervention studies. The study populations varied depending on the clinical trial, including overweight and obese adults, individuals of healthy weight, children with respiratory and gastrointestinal tract infections, and physically active men.	Various forms of mango and its derivatives:<br>• Fresh mango: 100 kcal/day for 12 weeks.<br>• Mango peel powder: 1 g twice daily for 84 days.<br>• Mango leaf extract (Zynamite®): 140 mg combined with quercetin.<br>• Mgf: 150 mg/day supplementation for 12 weeks.<br>• Fruit powder: 100 mg to 300 mg for 4 weeks.	↑ Nrf2<br>↓ROS<br>↓ COX-2/IL-6/TNF-α/PLA2<br>↑ AMPK/PI3K/AKT<br>↓ Glucose/lipids/appetite<br>↑Satiety/performance/endothelial function	IVb
[58]	Adult Albino Wistar rats with alloxan-induced type 1 diabetes.	Daily oral administration of mango peel ethanolic extract (200 mg/kg) or pure Mgf (20 mg/kg) for 21 days.	↓ Glucose<br>↑ Weight<br>↑ Glycogenesis<br>↑ Beta-cells<br>↑ Islets	IIId
[67]	A systematic review summarizing data from various sources, including human volunteers (Chinese males) and multiple animal species (rats, mice, rabbits, hamsters).	Reviews multiple interventions: for example, 150 mg/day for 12 weeks in human hyperlipidemia studies and various animal doses like 40 mg/kg for cardioprotection or 50 mg/mL for antioxidant comparison.	↓ Stress/inflammation/diabetes<br>↓ Radicals/cytokines	IVb
[53]	A systematic review analyzing 391 articles involving various in vitro cell lines (e.g., 3T3-L1, HepG2) and in vivo animal models (e.g., STZ-induced or db/db mice).	Synthesizes data on 1,073 isolated plant compounds, including Mgf, quercetin, and rutin, with varying doses.	↓ α-glucosidase<br>↓ Nephrotoxicity<br>↓ Glucose (↑ sensitivity, ↓uptake)	IVb
[48]	Male C57BL/6 mice (6-week-old) with metabolic disorders	Mgf at 150 mg/kg/day administered orally for 8 weeks (starting after 6 weeks of HFD).	↓ Weight<br>↓ Fat<br>↓ Glucose/lipids	IIId

Ref.	Study Model/Population	Intervention (concentration/Doses/ duration	Mechanism of Action	Level of evidence
	induced by a high-fat diet (HFD) for 14 weeks.		↓ M1/TNF-α<br>↑ Atg7/FGF21	
[60]	Male SD rats with Type II diabetes (STZ-induced) who received tibia bone implants.	Mgf was administered daily for a 3-month experimental period.	↓ Glucose/lipids<br>↑Repair/osteointegration(↑ BMP-2/RUNX2/osteoblasts,↓porosity/osteoclasts)	IIId
[103]	23 overweight and obese adults (15 men and 8 women). Age range 18–55 years (mean age ~29 for males and ~24 for females); Body Mass Index (BMI) between 27 and 40 kg/m². Generally healthy but with overweight or obesity; excluded if they smoked, had allergies to mangos or gluten, or used medications for blood pressure, lipids, or glucose.	Randomized crossover study with two dietary interventions and a 4-week washout period between them.<br>Snack 1 (Mango): 100 kcal of fresh sliced mangos (Tommy Atkins, Kent, or Haden cultivars), providing approximately 25 g of carbohydrates (22.3 g sugar, 2.6 g fiber).<br>Snack 2 (Control): 100 kcal isocaloric low-fat cookies (Nabisco Vanilla Wafers) served with 136 mL of water to match the water content of the mango.<br>Timing: Snacks were consumed within 5 minutes after an overnight fast.	↓ Glucosa (30′)<br>↓ Insulina (45′)<br>↑ Saciedad/plenitud<br>↓ hambre/ingesta/sed<br>↑ Adiponectina, CCK	II
[69]	Male Wistar rats (8-12 weeks old); experimental diabetes induced by streptozotocin (STZ) (70 mg/kg i.p.).	Mgf administered at a bolus dose of 40 mg/kg, followed by 20 mg/kg daily (i.p.) for 2 weeks.	↑ HRV<br>↓Oxidative/inflammatory stress(↓ TBARS/TNF-α, ↑ SOD)<br>↑ HIF-2α<br>↑ Chemosensing	IIId
[34]	In vivo model using male Wistar rats with induced prediabetes (via high-fat diet and low-dose streptozotocin) and in vitro enzyme assays.	Oral supplementation of Mango Peel Extract (MPE) at 5 g/kg of diet for 28 days following prediabetes induction.	↓ α-amilasa/α-glucosidasa<br>↓ insulina/HOMA-IR<br>↑ Glucosa<br>↓Lípidos (colesterol/VLDL/TAG)	IIIb
[50]	In vitro brown preadipocytes.	Thermogenic regulators (UCP1, PGC1α, PRDM16, PPARγ), mitochondrial biogenesis	↑ Thermogenesis<br>↑ Biogenesis/AMPK<br>↑ mtDNA/OCR	IVa

Ref.	Study Model/Population	Intervention (concentration/Doses/ duration	Mechanism of Action	Level of evidence
		markers (CIDEA, TFAM, SIRT1, NRF1), and AMPK phosphorylation.		
[80]	Male Sprague-Dawley rats with unilateral femoral artery ligation (acute hind limb ischemia-reperfusion injury) and streptozotocin-induced diabetes.	Ethanolic extract of mango seed kernels (EEMI), containing 0.5% Mgf, administered orally at 0.2 and 0.4 g/kg for 14 days.	↑ Recovery<br>↓ MDA/nitrite/cytokines<br>↑ GSH/IL-10<br>↑ eNOS/ICAM-1<br>↓ iNOS	IIId
[94]	Adult male Wistar rats with HFD-induced obesity.	Tea from Mangifera indica L. leaves (TML), Ubá variety, containing 0.717 mg/mL of Mgf. Consumption was 24.7 mL/day for 8 weeks.	↓ Fat<br>↑ IL-10<br>↓ TNF-α<br>↑ PPAR-γ/LPL<br>↓ FAS	IIId
[109]	In vitro HAEC cells and in vivo male New Zealand White rabbits.	Ketone Molecule (KM) encapsulated in pea protein nanoparticles using Mgf (from mango peel) as a natural crosslinking agent.	↑ AUC<br>↑ Bioavailability	III d
[104]	*Sphaeranthus indicus* and Mangifera indica blend in humans 40 healthy, physically active men (recreational athletes), aged 18 to 40, with at least 6 months of resistance training experience.	Daily oral supplementation with 650 mg of a proprietary blend (LI12542F6) of *S. indicus* flower and M. indica bark extracts (in a 2:1 ratio) or a placebo, for 56 consecutive days, combined with a 4-day-per-week resistance training program.	↑ Strength/endurance/size<br>↑ Testosterone<br>↓ Cortisol<br>↑ mTOR/eNOS<br>↑ Synthesis/mitochondria	II
[35]	In vitro bioassay using α-glucosidase from *S. cerevisiae* and In silico molecular docking simulations using 3A4A and 3TOP receptors.	Mgf isolated from *Artabotrys sumatranus* leaf extract.	↓ α-glucosidase (83.72)<br>↑ Binding<br>↓ Better than acarbose	IIIf
[62]	Swiss albino mice with Alloxan-induced	Oral administration of hydro-alcoholic extract of *M. indica* cv.	↓ Glucosa<br>↑ Lípidos<br>↓ Daño pancreático (estrés oxidativo)	IIId

Ref.	Study Model/Population	Intervention (concentration/Doses/ duration	Mechanism of Action	Level of evidence
	diabetes.	Anwar Ratol leaves at 550, 750, or 950 mg/kg daily for 7 days.		
[39]	In vitro biochemical assays using porcine pancreatic α-amylase and yeast α-glucosidase.	Nine synthetic xanthones and commercial parents: Mgf (4), α-mangostin (5), and γ-mangostin (6) at concentrations up to 200 μM.	⬇ Amylase/glucosidase	IVa
[95]	Albino Wistar rats with STZ-nicotinamide (NA)-induced diabetes.	Oral administration of Mgf at 40 mg/kg/day for 30 days.	⬇ Glucose/HbA1c<br>⬆ PPARγ/FALDH	IIId
[87]	In vitro L6 myotubes (rat skeletal muscle cells) under oxidative stress induced by 4-hydroxy-2-nonenal (HNE).	Mgf(20 μM) for 24 hours.	⬇ ROS/MDA/carbonyls<br>⬇ Damage<br>⬆ Genes<br>⬆ Glucose uptake	IVa
[92]	In vitro study using the 3T3-L1 murine preadipocyte cell line.	In vitro study using the 3T3-L1 murine preadipocyte cell line.	⬇ Lipids/α-glucosidase<br>⬆ GSH/adiponectin<br>⬆ HO-1/PPARγ	IVa
[19]	Female C57BL/KsJdb/+ (db/+) mice, used as a genetic model for Gestational Diabetes Mellitus (GDM).	Mgf was administered at 50 mg/kg/day by oral gavage starting before mating and continuing until gestation day 18.	⬇ Glucose<br>⬇ ER stress/cytokines<br>⬆ Fetal outcomes	IIId
[52]	In vivo model using male albino rats with streptozotocin (STZ)-induced diabetes and in silico molecular docking.	Isolated Mgf (SA1) administered orally at 50 and 100 mg/kg daily for 15 days.	⬇ Glucose/oxidative stress/lipids<br>⬆ Weight/glycogen<br>⬆ PPARγ/GLUT4	IIIc
[54]	Male C57BL/6 mice with STZ-induced diabetic nephropathy.	Oral Mgf at 15, 30, or 60 mg/kg/day for 4 weeks.	⬇ Dysfunction/fibrosis<br>⬇ PI3K/Akt<br>⬇ TGF-β1/ECM<br>⬆ PTEN	IIId
[74]	Male albino Wistar rats (150–200 g); diabetes induced by STZ (70 mg/kg i.p.) followed by induced myocardial	Mgf (40 mg/kg/day; i.p.) for 28 days.	⬆ Function/architecture<br>⬇ AGE-RAGE/JNK/p38<br>⬆ ERK1/2<br>⬇ Stress/inflammation/apoptosis	IIId

Ref.	Study Model/Population	Intervention (concentration/Doses/ duration	Mechanism of Action	Level of evidence
	ischemia-reperfusion (IR) injury.			
[56]	Adult male Wistar rats fed a high-fat diet (HFD) for 10 weeks and given STZ to induce diabetes and metabolic syndrome.	Oral Mgf at 40 mg/kg/day from the 5th to the 10th week.	⬇ DPP-IV<br>⬇ Glucose/lipids/inflammation<br>⬇ CPK-MB	IIId
[57]	Wistar rats with experimentally induced diabetes and metabolic syndrome (HFD + STZ).	Oral Mgf (40 mg/kg) as monotherapy or in combination with Metformin (100 mg/kg) from the 4th to the 10th week.	⬇ IR<br>⬇ Apoptosis/oxidative stress/inflammation<br>⬆ β-cells	IIId
[38]	In silico molecular docking/dynamics and in vitro enzymatic assays using porcine α-amylase and yeast α-glucosidase.	Mgf and Friedelin; tested for stimulatory/inhibitory activity at concentrations up to 50 μM (for GCK) and 16 mg/mL (for other enzymes).	⬆ GK<br>⬇ DPP-IV/α-amilasa/α-glucosidasa	IIIf
[88]	In silico prediction (IT Microcosm system) and in vivo Sprague–Dawley rats (inflammation/diabetes) and *Mus musculus* mice (hypercholesterolemia).	Mgf isolated from *Hedysarum neglectum* (at least 95% purity) at doses of 50 and 100 mg/kg daily for 7 to 14 days.	In silico: ⬇ inflammation/diabetes<br>In vivo: ⬆ glucose/lipids	IIIc
[106]	Male Kunming mice fed a high-fat diet (HFD) to establish a model of NAFLD.	Intraperitoneal injection of Mgf at 15, 30, or 60 mg/kg/day for 12 weeks.	⬇ NF-κB/JNK<br>⬆ Autophagy (⬆LC3/ ⬇p62)<br>⬆ Insulin signaling	IIId
[42]	In vivo model using 12-month-old male C57BL/6J mice subjected to partial pancreatectomy (PPx) and in vitro primary culture of mouse islet β-cells.	Intraperitoneal administration of Mgf at 90 mg/kg daily for 28 consecutive days.	⬇ Glucose<br>⬆Tolerance/insulin/islets/proliferation<br>⬇ Apoptosis<br>⬆ Cyclins/cdk4<br>⬇ p16/p27	IIIb

Ref.	Study Model/Population	Intervention (concentration/Doses/ duration	Mechanism of Action	Level of evidence
[18]	In vitro porcine pancreatic lipase and in vivo male C57BL/6J mice with HFD-induced obesity.	Oral administration of Mgf (200 mg/kg/day) for 12 weeks.	↑ Lipase<br>↑ Excretion 52.5%<br>↓ Lipase<br>↓ Absorption<br>↓ Lipids	IIIb
[102]	Male Wistar rats; hyperglycemia was induced via streptozotocin (STZ).	NSC-Mgf nanoparticles (Mangiferin loaded in N-succinyl chitosan-alginate) administered orally (10 mg/kg/day) for 28 days.	↑ Solubilidad<br>↓ Glucosa/colesterol/triglicéridos	IIId
[63]	In vitro cell lines (HUVECs, L929) and in vivo male SD rats with full-thickness wounds infected by *S. aureus*.	Rh@Ag-MFG hydrogel (self-assembled Mgf hydrogel loaded with rhein-functionalized silver nanoparticles) applied topically.	↑Hemostasis<br>↓ Infection/oxidation	IIIb
[15]	A meta-analysis of 19 animal articles (including rats, mice, and hamsters) with a total of 535 animals.	Interventions involved oral Mgf with doses ranging from 5 to 422 mg/kg for durations between 11 days and 16 weeks.	↓ Glucose<br>↑ Weight / obese<br>↓ TC/TG	I
[85]	In vivo: ICR mice fed a high-fat diet. In vitro: Isolated rat PVAT and 3T3-L1 adipocytes	In vivo: 50 mg/kg/day orally for 2 weeks. In vitro: Pretreatment with 0.1, 1, or 10 μM Mgf.	↓ ER stress/NLRP3<br>↑ LKB1/AMPK<br>↑ NO	IIIb
[68]	Sprague-Dawley rats and ICR mice fed a high-fat diet, along with perivascular adipose tissue (PVAT) samples.	In vivo: Mice were fed HFD for 2 weeks with daily Mgf (50 mg/kg, oral). In vitro: PVAT was pretreated with Mgf (0.1–10 μM).	↓ IR/ER stress/NLRP3/ROS<br>↑ LKB1/AMPK<br>↑ p-Akt/eNOS	IIIb
[97]	In vitro HepG2 human liver cells under a high-fat environment induced by palmitic acid (PA).	Combination of Mgf and Epigallocatechin gallate (EGCG) at an optimum concentration of 25:50 μM for 24 hours.	↓ Hyperlipidemia<br>↑ Oxidation<br>↓ TG/TC<br>↑ AMPK/PPARα	IVa
[51]	In vitro study using differentiated 3T3-L1 adipocytes.	Mgf at 0.1, 1, and 10 μmol/L during 8 hours of hypoxic incubation (1% O2).	↓ HIF-1α/lactate/cytokines<br>↑ Akt/GLUT4<br>↓ NF-κB<br>↑ AMPK	IVa
[64]	An infected diabetic wound model in rats	Local application of a glycoengineered EV-based	↑ Nrf2<br>↓ Oxidative stress	IIIb

Ref.	Study Model/Population	Intervention (concentration/Doses/ duration	Mechanism of Action	Level of evidence
	and in vitro endothelial cells and fibroblasts.	biohybrid nanorobot (MF@DeMEV/SA-MNP) loaded with Mgf, driven by a magnetic field.	↑ Angiogenesis/collagen<br>↑ Healing	
[81]	Male Sprague-Dawley rats with hyperuricemia induced by potassium oxonate and in vitro HUVECs (human umbilical vein endothelial cells).	Mgf administered at 30, 60, or 120 mg/kg/day intragastrically for 12 weeks. In vitro HUVECs were treated with Mgf (75–300 μM).	↓ Hypertension<br>↓ CRP/ICAM-1<br>↑ NO<br>↓ Oxidative stress	IIIb
[86]	In vivo: Male C57BL/6J mice (5 weeks old). A model of Non-alcoholic fatty liver disease (NAFLD) was induced by feeding the mice a high-fat diet (HFD) for 12 weeks.<br>In vitro: HepG2 cells (human hepatoma cell line).	Compound: Mgf, a natural carbon glycoside.<br>Animal Doses: Three doses administered daily via oral gavage (i.g.):<br>Low: 25 mg/kg/day.<br>Middle: 50 mg/kg/day.<br>High: 100 mg/kg/day.<br>Animal Duration: 12 weeks.<br>Cell Concentrations: Various concentrations, notably 25 μM, 50 μM, and 100 μM. Free fatty acid (FFA) induction was performed using 300 μM PA-BSA.	↑ AMPK<br>↑ Glucose/oxidation<br>↓ NLRP3<br>↓ IL-1β<br>↓ Weight/lipids/ALT/AST/IR<br>↑ Tolerance<br>↓ Steatosis	IIIb
[65]	Female Sprague-Dawley rats; PCOS model induced by letrozole (LET) combined with a high-fat diet (HFD).	Mgf at low-dose (50 mg/kg/d) or high-dose (200 mg/kg/d) for 4 weeks.	↓ Insulin resistance<br>↓ LH/FSH<br>↓ Anti-apoptotic activity<br>↑ Cytc, Blautia, Coprococcus	IIId
[91]	In vitro model based on two specific cell lines:<br>HepG2: Human hepatoma cells (liver).<br>C2C12: Mouse myoblasts differentiated into myotubes (skeletal muscle).<br>An insulin resistance (IR) model was established by inducing	Mgf 12.5, 25, and 50 μM., and palmitic acid induction (0.25 mM) were performed simultaneously for 24 h.	↑Uptake/GLUT2-4/PPARα/CD36/CPT1<br>↓ TG/FFA; siRNA PPARα<br>↑ Mechanism: PPARα, Oxidation<br>↓ Lipids<br>↑ Insulin	IVa

Ref.	Study Model/Population	Intervention (concentration/Doses/ duration	Mechanism of Action	Level of evidence
	the cells with palmitic acid (PA) to simulate lipid accumulation and metabolic stress.			
[45]	Male KK-Ay mice (a model for type-2 diabetes and obesity).	Mango leaf 70% ethanol extract (ME) administered orally at doses of 200 to 500 mg/kg/day for a duration of 8 weeks.	↓ Fat/lipids<br>↓ ACC/FAS PPARγ<br>↑ AMPK	IIId
[99]	Channel catfish (*Ictalurus punctatus*), primary catfish hepatocytes, and NCTC 1469 mouse hepatocytes.	High-starch diet (HCD) supplemented with MGF at 100 or 500 mg/kg for 8 weeks. In vitro cells were treated with 1.25 μM MGF.	↓ Injury/lipids<br>↓ Vía Srebf2/sp1	IIIb
[83]	In vivo: Rats with streptozotocin (STZ)-induced diabetic nephropathy.	Treatment with Mgf monosodium salt.	↑ GPX4/FSP1<br>↓ ACSL4<br>↓ MAPK/NF-κB<br>↓ Ferroptosis<br>↓ Inflamation<br>↑ p-IRS1/PI3K/Akt<br>↓ IR	IIId

The hierarchical classification of the studies is based on the following level of evidence: I. Very high, with Meta-analyses and randomized controlled clinical trials; II. High, with clinical trials; IIIa. Moderately strong, with comprehensive clinical evidence (In silico + In vitro + In vivo); IIIb. Moderately strengthened, with mechanistic studies with systemic validation (In vitro + In vivo); IIIc. Moderately strengthened, with molecular prediction + physiological effect (In silico + In vivo); IIId. Moderate, with physiological validation in an animal model (In vivo); IIIe. Very low, strengthened by molecular prediction with single-cell effect (In silico + In vitro) or IIIf (In vitro+In silico); IVa. Low, with cellular mechanistic evidence (In vitro); IVb. Low, narrative review or network pharmacology, V. Very low, with predictive evidence without biological validation (In silico).
